# Grapevine Diversity and Genetic Relationships in Northeast Portugal Old Vineyards

**DOI:** 10.3390/plants10122755

**Published:** 2021-12-14

**Authors:** Diana Augusto, Javier Ibáñez, Ana Lúcia Pinto-Sintra, Virgílio Falco, Fernanda Leal, José Miguel Martínez-Zapater, Ana Alexandra Oliveira, Isaura Castro

**Affiliations:** 1 Department of Genetics and Biotechnology, University of Trás-os-Montes e Alto Douro, 5000-801 Vila Real, Portugal; dianah@utad.pt; 2 Institute of Grapevine and Wine Sciences (ICVV), Spanish National Research Council (CSIC), University of La Rioja and Government of La Rioja, 26007 Logroño, Spain; javier.ibanez@icvv.es (J.I.); zapater@icvv.es (J.M.M.-Z.); 3 Centre for Research and Technology of Agro-Environmental and Biological Sciences (CITAB), Institute for Innovation, Capacity Building and Sustainability of Agri-Food Production (Inov4Agro), University of Trás-os-Montes e Alto Douro, 5000-801 Vila Real, Portugal; asintra@utad.pt (A.L.P.-S.); fleal@utad.pt (F.L.); anaolive@utad.pt (A.A.O.); 4 Chemistry Research Centre, Vila Real (CQ-VR), University of Trás-os-Montes e Alto Douro, 5000-801 Vila Real, Portugal; vfalco@utad.pt

**Keywords:** chlorotype, genotyping, grape germplasm, kinship relationship, population structure, SNP, SSR, *Vitis vinifera* L.

## Abstract

More than 100 grapevine varieties are registered as suitable for wine production in “Douro” and “Trás-os-Montes” Protected Designations of Origin regions; however, only a few are actually used for winemaking. The identification of varieties cultivated in past times can be an important step to take advantage of all the potential of these regions grape biodiversity. The conservation of the vanishing genetic resources boosts greater product diversification, and it can be considered strategic in the valorisation of these wine regions. Hence, one goal of the present study was to prospect and characterise, through molecular markers, 310 plants of 11 old vineyards that constitute a broad representation of the grape genetic patrimony of “Douro” and “Trás-os-Montes” wine regions; 280 samples, grouped into 52 distinct known varieties, were identified through comparison of their genetic profiles generated via 6 nuclear SSR and 43 informative SNP loci amplification; the remaining 30 samples, accounting for 13 different genotypes, did not match with any profile in the consulted databases and were considered as new genotypes. This study also aimed at evaluating the population structure among the 65 non-redundant genotypes identified, which were grouped into two ancestral genetic groups. The mean probability of identity values of 0.072 and 0.510 (for the 6 SSR and 226 SNP sets, respectively) were determined. Minor differences were observed between frequencies of chlorotypes A and D within the non-redundant genotypes studied. Twenty-seven pedigrees were confirmed and nine new trios were established. Ancestors of eight genotypes remain unknown.

## 1. Introduction

The wine denominations “Douro”, the oldest demarcated and regulated winemaking region in the world, and “Trás-os-Montes”, represent approximately 29% of the Portuguese vineyard area for wine production (22 and 7%, respectively) [1]. These Protected Designations of Origin (PDO) situated in Northeast Portugal have an ancient and diverse viticulture history and they are characterised by their mountains with steep slopes and valleys propitious to the existence of distinct microclimates, which led to the evolutionary need of grapevine adaptation to different conditions [2]. In this sense, traditional viticultural practices and local climates were crucial to the high genetic diversity observed in these wine regions. On the other hand, the referred factors also led to the appearance of a large number of synonyms (different names for the same variety) and homonyms (common name for different varieties) among Portuguese grapevines, which make difficult the identification of true genotypes. Hence, accurate identification of true-to-type *V. vinifera* samples is the first prerequisite for the correct management of germplasm collections, selection of suitable parents for breeding programs, and clarification of synonyms, homonyms, and naming inaccuracies among grape varieties [3,4,5,6].

Identification of grape varieties has been generally based on the phenotypic traits of vegetative and reproductive structures, but morphological features are not sufficiently reliable for the classification of closely related varieties due to genotype–environment interactions [7]. Moreover, the global exchange of clonally propagated materials over time, across distinct geographic and edaphoclimatic regions has challenged the morphological identification of grape genotypes [8]. In this sense, molecular characterisation, mainly through microsatellites (or Simple Sequence Repeats, SSRs) and Single Nucleotide Polymorphisms (SNPs), is the favoured way to characterise diversity within a germplasm repository for varietal identification [9,10,11,12,13]. 

SSR markers, because of their high degree of polymorphism, reproducibility, and co-dominant nature, have become the markers of choice for exchange of information concerning grapevine genetic resources [12]. Several studies report on the genetic diversity among Portuguese grape varieties based on nuclear and/ or chloroplastidial SSR loci (nSSR and cpSSR) [3,4,5,14,15,16,17,18]. Moreover, the combination of three cpSSR markers are informative enough to distinguish the four different major chloroplastidial haplotypes or chlorotypes (maternally inherited) in *V. vinifera* [19]. Chlorotypes have been useful to show the multiple origins of *V. vinifera* spp. *sativa* (cultivated grapevine), by analysing their distribution in local *V. vinifera* spp. *sylvestris* and *sativa* [13], and to follow the maternal line of any variety, including determining the female progenitor in a pedigree.

At the present time, SNP markers are considered one of the most powerful tools recently developed [10,13,20]. Although they have a biallelic nature, which reduces their information content when compared with SSR loci, SNPs are the most abundant DNA sequence polymorphisms widespread in a plant genome; thus, it is possible to multiplex hundreds or thousands of loci in one chip and, in a single analysis, to evaluate allelic variations throughout the entire genome. Locus availability and, consequently, the possibility to retrieve thousands or millions of SNP from genome sequences has revolutionised plant genomic research over the last decade. In addition, SNP data can be compared across different platforms and laboratories more easily than microsatellite data, since normalisation with reference varieties is not required; hence, this higher reproducibility facilitates the integration and interpretation of genotyping data throughout grape genebanks and databases [21]. Another advantage of SNP genotyping is its automatization, as sample analyses may be completely automated in high-throughput research programs [22].

The sequence of the grapevine reference genome is available [23,24] and can be used to draw information on genes of interest. Programmes of sequencing and resequencing of the grape genome have generated a database including an extensive number of SNPs, useful for setting up different genotyping SNP panels [7,25,26,27,28]. A reference SNP database is that of the Instituto de Ciencias de la Vid y del Vino (ICVV), which includes data from many cultivated and wild grapevine samples from diverse geographic regions, genotyped for 48 SNP markers that had been validated for varietal characterisation [29].

These SNP markers are used to investigate genetic variability, discriminating among wild and cultivated *V. vinifera* populations, to infer genetic structure, and to identify kinship relationships [7,27,28,29,30,31,32,33,34,35,36,37,38]. Characterisation studies of the Portuguese grapevine (*V. vinifera* subsp. *vinifera* and *sylvestris*) germplasm were also been undertaken, using the ICVV sets of 48 [6] and 261 SNPs [20]. Currently the *Vitis* International Variety Catalogue (*V*IVC, www.vivc.de; accessed on 2 July 2021) includes SNP data of 112 markers for many varieties [39].

As a result of changes in worldwide wine consumption and European Union incentives [40], radical transformations in European viticulture have occurred in the last decades, namely vineyard restructuring and conversion to commercially available clones from a reduced number of grape varieties. Consequently, crop vulnerability to several abiotic and biotic stresses has increased, producing a massive negative impact on the rich heritage in grape varieties, so crucial to an environmentally sustainable viticulture. A possible response towards these projected future stresses in vineyards in a world with high-demanding wine consumers is to preserve a wider range of grape varieties. This action thus demands a continuous grape varietal prospection for their conservation and putative exploitation in traditional winemaking locations, since they possess old genetic resources on the edge of extinction. In this sense, the main goals of this study were the: (i) molecular identification in old traditional vineyards of a broad representation of grapevine patrimony of “Douro” and “Trás-os-Montes” regions contributing to deepen the knowledge of Northeast Portugal grapevine gene pool. Varietal discrimination was carried out by using the set of six microsatellite markers recommended by OIV [41] and the 48 SNP set developed by Cabezas et al. [29] and comparing SSR and SNP profiles obtained with those of the *V*IVC database and the ICVV-SNP database, respectively; (ii) evaluation of genetic diversity and relationships among the grape genotypes detected, through SSR and SNP (240 SNP) markers; (iii) determination of chlorotypes and their frequencies through cpSSR and SNP loci amplification; and (iv) determination of first-order kinship relationships among grape varieties using the 240-SNP set.

## 2. Results and Discussion

**Figure 1:**
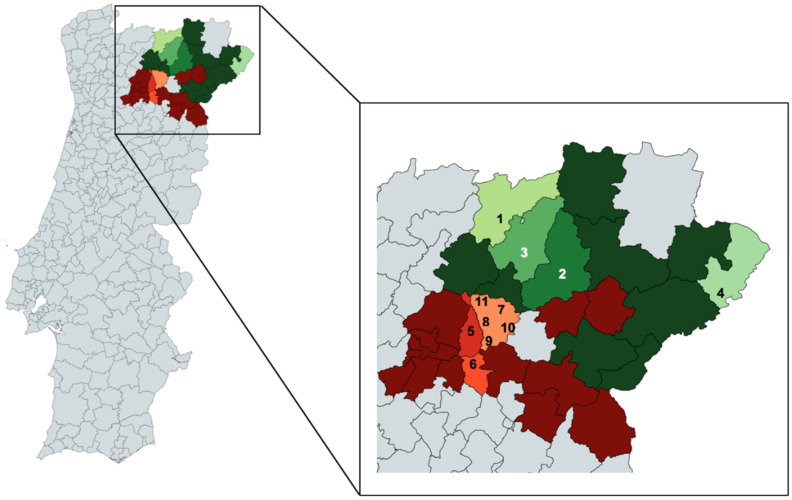

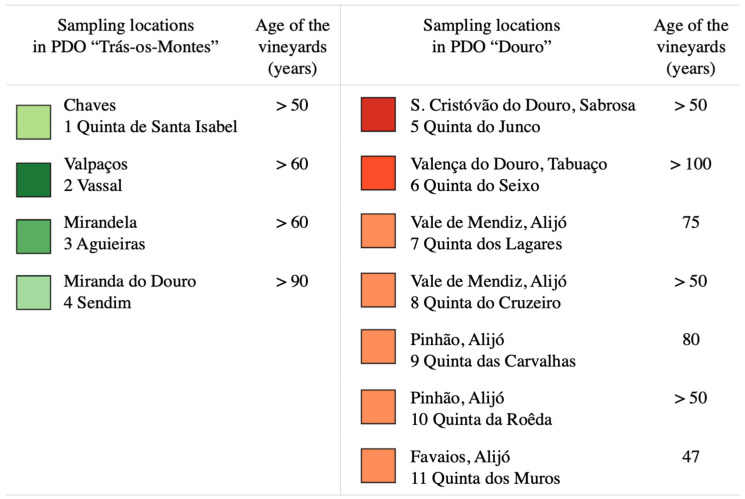
Geographical distribution of 310 samples in 11 locations of “Douro” and “Trás-os-Montes” PDO regions in Northeast Portugal and the age of vineyards (created with Map Chart).

A total of 310 grapevines were sampled in traditional vineyards throughout “Douro” and “Trás-os-Montes” PDO regions (Figure 1). These vines (older than 47 years) were located in old vineyards of wine-growing companies or in small vine parcels, such as the Vassal, Aguieiras, and Sendim sampling locations, belonging to local wine producers for self-consumption.

Samples were characterised using molecular markers, namely Simple Sequence Repeats (SSR) and Single Nucleotide Polymorphisms (SNP), starting by varietal identification and followed by population structure, genetic diversity, and pedigree analyses with non-redundant genotypes.

All samples were initially analysed with six nuclear microsatellites (nSSR; approved as descriptors by the OIV [41]) and three chloroplastidial microsatellites (cpSSR) [19]. Microsatellite markers are most adapted for varietal identification (fast results, easy to assay, cost effective, and available databases). However, SSR genotyping is subject to technical variations that required calibration between laboratories [12]. 

An SNP array was first proposed as an alternative to SSR for varietal identification by Cabezas et al. [29]. These authors reported this 48-SNP set with a discrimination power similar to 14–16 microsatellite markers. Several research groups are currently using the 48-SNP set previously defined for that purpose [6,7,29,42]. Thus, non-redundant grape varieties identified and all non-identified samples, through SSR genotyping, were also profiled with the 48-SNP array selected by Cabezas et al. [29].

Non-redundant genotypes for the 48-SNP set were then genotyped with 192 additional SNP loci for subsequent analyses of population structure, genetic diversity, and parentage relationships. However, only 226 out of 240 SNP markers were informative; 14 SNPs were discarded, because data produced by 11 SNP were missing in at least 61% of the samples, and three SNP were monomorphic in the genotypes analysed.

### 2.1. Genetic Identification Based on nSSR and SNP Markers

**Figure 2:**
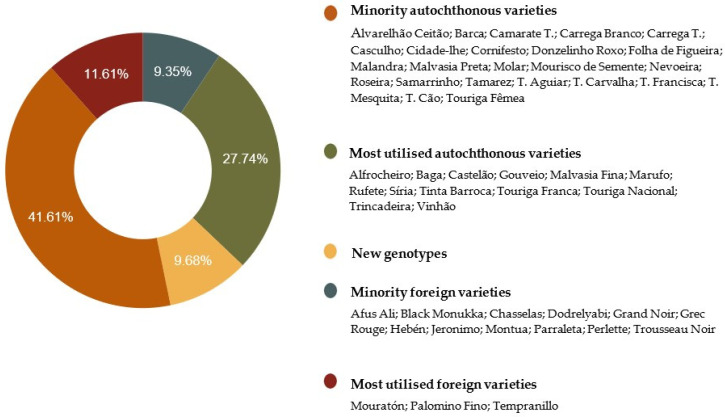
Distribution of the studied grapevine varieties in PDO “Douro” and PDO “Trás-os-Montes”, according to their vineyard Table 1. Fifty-two genotypes were identified belonging to either the most cultivated varieties in Portugal (which means a representation superior to 1% of total area) or to the minority varieties group. Thirteen new genotypes were also detected.

Two hundred and eighty samples were identified through comparison of their genetic profiles generated via six nuclear SSR and 43 informative SNP loci amplifications. The SSR and SNP profiles were compared to those stored in the *V*IVC and ICVV-SNP databases, respectively. The *V*IVC database includes 5424 genetic profiles [39] and the ICVV-SNP database more than 2800 non-redundant genotypes for 48 SNPs of diverse genetic and geographic origins. Fifty-two distinct grapevine varieties were detected, from which 37 were described as autochthonous to Portugal (Table 1; Figure 2; Supplementary Tables S1–S4) [3,4,6,17,20]. The SSR and SNP profiles also allowed the identification of 15 foreign genotypes, which corresponded to varieties from Spain (‘Hebén’, ‘Jeronimo’, ‘Montua’, ‘Mouratón’, ‘Palomino Fino’, ‘Parraleta’, and ‘Tempranillo’), France (‘Chasselas’, ‘Grand Noir’, ‘Grec Rouge’, and ‘Trousseau Noir’), Georgia (‘Dodrelyabi’), Lebanon (‘Afus Ali’), and the United States of America (‘Black Monnuka’ and ‘Perlette’). According to *V*IVC data, the varieties ‘Hebén’, ‘Mouratón’, ‘Montua’, ‘Tempranillo’, ‘Palomino‘, ’Parraleta’, ‘Grec Rouge’, and ‘Trousseau Noir’ are synonyms of the Portuguese varieties ‘Mourisco Branco’, ‘Tinta Gorda’, ‘Diagalves’, ‘Aragonez’, ‘Malvasia Rei’, ’Tinta Caiada’, ‘Rabigato Francês’, and ‘Bastardo’, respectively (Table 1). Portuguese grapevine germplasm has been recently referred as the main route for gene flow from Iberian Peninsula to Western and Central Europe [43]. The variety ‘Hebén’ (syn. ‘Mourisco Branco’ in Portugal and with great importance in the genetic network of Iberian Peninsula grapevine varieties [20]) is one of the few evidences of the presence of Iberian varieties in Italy, specifically, in the island of Sardinia, being the genitor of three local varieties [44]. 

The remaining 30 samples, accounting for 13 different SSR and SNP genetic profiles, did not match with any profile stored in the *V*IVC and ICVV-SNP databases, respectively, and are being further studied (Table 1; Figure 2; Supplementary Tables S3 and S4). Their discovery uncovers once again the richness of the Portuguese gene pool as already highlighted by Cunha et al. [6]. Most likely, these unique genotypes correspond to minor autochthonous varieties from Portugal, since the lack of profile matching with international databases, and the fact that each genotype (even with more than one sample identified) has been found in a single sampling location (Table 1). Maraš et al. [38] used the term “proto-varieties” to designate plants that have been only cultivated by local grape growers, i.e., plants that directly grow from seeds, or have been multiplied through cuttings just once, from the place where the seed germinated to the orchard. Eventually, they could be multiplied and distributed, becoming varieties. This is how most of the varieties were originated in the past, but these traditional techniques are not used anymore in Western European regions. 

The most commonly observed genotype among the 280 identified grapevine samples corresponded to the variety ‘Trincadeira’, which was found 28 times, followed by ‘Casculho’ (23), ‘Mouratón’ and ‘Síria’ (17), ‘Roseira’ (16), ‘Marufo’ (13), ‘Tempranillo’ (13), ‘Camarate Tinto’, ‘Carrega Branco’, and ‘Malvasia Preta’ (11), and ‘Trousseau Noir’ (10). These most commonly found genotypes across the sampled old vineyards may reflect their relevance in the past century.

Sixteen identified genotypes (‘Afus Ali’, ‘Alfrocheiro’, ‘Alvarelhão Ceitão’, ‘Baga’, ‘Carrega Tinto’, ‘Cidadelhe’, ‘Grand Noir’, ‘Grec Rouge’, ‘Jeronimo’, ‘Malvasia Fina’, ‘Molar’, ‘Parraleta’, ‘Rufete’, ‘Samarrinho’, ‘Tamarez’, and ‘Tinta Mesquita’) and eight non-identified genotypes (NG002, NG003, NG004, NG005, NG006, NG010, NG011, and NG012) were found only once in the study (Table 1; Supplementary Table S1). 

The selection of plants was based on the difficulty of their morphological identification by ampelographers, where only 31 out of 310 samples were previously named but mainly with local names (Supplementary Table S2). Microsatellite and SNP loci amplification allowed the confirmation of the varietal identity of six samples (‘Roseira’, ‘Trousseau Noir’ syn. ‘Bastardo‘, ’Casculho’, ‘Tinta Francisca’, ‘Grand Noir’ and ‘Rufete’). On the other hand, samples with different names but which fully matched at the genotyped nSSR and SNP loci were also found and they were considered synonyms or misnomers. The ’Verdelheira’ sample was identified as the Portuguese variety ‘Gouveio’; this variety is known as ‘Verdelho’ in the Dão wine region [6] and it has ‘Godello’ (or ‘Verdello2′) as synonyms in Galicia [45]. Other synonyms were established, namely ‘Tinta Amarela Antiga’, ‘Sousão’, and ‘Rabigato Francês’ samples whose genetic profiles match with those of ‘Trincadeira’, ‘Vinhão’, and ‘Grec Rouge’ varieties, respectively (Supplementary Table S2).

Moreover, ‘Mourisco’ and ‘Bastardinho’ samples share the same nSSR and SNP profiles, but were revealed to be the ‘Mouratón’ (syn. ‘Tinta Gorda’) genotype described by Cunha et al. [6]. ‘Mourisco de Semente’, ‘Mesquita’, ‘Tinta Carvalha’, and ‘Cornifesto’ samples also produced identical genetic profiles, which were found to match with the ‘Roseira’ variety profile and represent misnaming cases (Supplementary Table S2). The ‘Tinta Grossa’ sample revealed a different genetic profile from the official variety ‘Carrega Tinto’ but matched with that of the Portuguese variety ‘Casculho’. ‘Tinta do Bragão’ sample had an identical genotype to ‘Tinta Mesquita’ variety; hence, this sample was misnamed, since it is different from its official variety name ‘Barreto’ [3,6]. Other cases of wrong denominations are those of ‘Preto Martinho’, ‘Touriga Fêmea’, ‘Tinta Bairrada’, ‘Barca’, ‘Moreto’, and ‘Tinta Malandra’ samples, whose genetic profiles matched to the ‘Cidadelhe’, ‘Touriga Nacional’, ‘Baga’, ‘Marufo’, ‘Camarate Tinto’, and ‘Touriga Franca’ genetic profiles, respectively (Supplementary Table S2). Despite ‘Juan García’ being described as a ‘Mouratón’ variety in Martín et al. [46] and Díaz-Losada et al. [47], the ‘João Garcia’ sample was revealed to be the ‘Touriga Franca’ variety (Supplementary Table S2). The ‘Loulela’ and ‘Polita’ samples were identified as ‘Folha de Figueira’ and ‘Síria’ varieties, respectively; there is no reference in the literature to these local names being synonyms with the referred grape varieties.

The genetic profiles of ‘Mourisco’, ‘Mourisco de Semente’, ‘Lázaro’, and ‘Rosada’ samples, generated through nSSR and SNP analyses, did not match with any sample profile included in the *V*IVC and ICVV-SNP databases. This fact led to the conclusion that the two first samples were incorrectly named, but further analysis of the other two samples is needed to clarify if they are a situation of naming inaccuracy. Nevertheless, all four samples were considered as new genotypes (Supplementary Table S2).

Regarding the use of the berry, 36 out of the 52 genotypes with varietal identification corresponded to wine varieties (69.3%), one to a table variety (1.9%), 14 to varieties with a double wine/table use (26.9%), and one to a variety with double table/raisin use (1.9%; Table 1).

There are 343 authorised varieties for wine production in Portugal [48]. All varieties detected in this work are included on the list of varieties authorised for winemaking, except ‘Afus Ali’, ‘Black Monukka’, ‘Dodrelyabi’, ‘Jeronimo’, and ‘Perlette’. More particularly, 42 out of the identified 47 grape varieties authorised for winemaking in Portugal are also approved for red and white wine production under PDO “Douro” and “Trás-os-Montes” (Table 1) [1,49,50]. Not on the lists of grape varieties suitable for the production of wine in these PDO regions are three autochthonous (‘Camarate Tinto’, ‘Donzelinho Roxo’, and ‘Molar’) and six foreign (‘Afus Ali’, ‘Black Monukka’, ‘Dodrelyabi’, ‘Grec Rouge’, ‘Jeronimo’, and ’Perlette’) varieties. Nonetheless, the varietal diversity found in the study is not entirely exploited, since only 39.35% of the Portuguese native and foreign varieties found are widely cultivated in these PDO wine regions (Figure 2) [1].

Another fact to take into account was that seven red varieties (‘Cornifesto’, ‘Marufo’, ‘Rufete’, ‘Tinta Barroca’, ‘Tinta Francisca’, ‘Tinto Cão’, and ‘Vinhão’) and five white (‘Diagalves’, ‘Gouveio’, ‘Malvasia Rei’, ‘Samarrinho’, and ‘Síria’) listed as suitable for production of PDO wines are described as late-maturing ones [51,52]. Hence, high quality PDO wines from late-maturing grape varieties will likely need to be considered under a future warmer climate, to cope with the extreme hot temperatures and precipitation deficits registered worldwide, and especially in Portugal.

### 2.2. Nuclear SSR and SNP Diversity

SNP and SSR profiles were compared to assess the genetic diversity of the 65 non-redundant genotypes and these results are summarised in Table 2 (and Supplementary Table S5). A total of 57 alleles were obtained at the OIV set of six microsatellite loci ranging from 7 (VVMD27) to 12 (VVS2) and with an average of 9.5 alleles per locus. The level of polymorphism found was comparable with that reported for other *V. vinifera* germplasm diversity studies assessed with SSR markers. An analysis of 51 varieties using the six OIV loci described a range of 7 (VVMD27) to 11 (VVS2) alleles, with an average of 8.2 [17]. The nuclear microsatellite diversity study of 57 grape varieties reported by Cunha et al. [53] showed a total of 53 alleles scored across the six loci, 13 alleles for the VVS2 locus, and 8 for all the others, with an average of 8.8 alleles per locus. Moreover, an analysis of 39 Portuguese varieties described by Castro et al. [4], also using the OIV set of six loci, showed that the allele number ranged from 6 (VVMD27) to 10 (VVMD5 and VVS2), with a mean value of 8.3 alleles per locus.

The allele size varied between 132 (VVS2) and 263 bp (VVMD7). VVMD7-239 and ssrVrZAG62-191 alleles were the most frequent, with over 43% of frequency. On the other hand, 25 alleles (43.86%) showed a frequency lower than 5%, eight of them being unique alleles (Supplementary Table S5). Specific alleles were identified in ‘Grand Noir’ (VVS2-138), ‘Baga’ (VVS2-154), ‘NG012’ (VVMD7-237), ’Dodrelyabi’ (VVMD7-255), ‘NG011’ (VrZAG62-205), ‘Grec Rouge’ (VrZAG79-237), ‘NG013’ (VrZAG79-247), and ‘Perlette’ (VrZAG79-253); (Supplementary Table S5).

Allele frequencies and genetic parameters were also determined for the 226 SNP set (Table 2). The minor allele frequency (MAF) was analysed, since it is a measure of the discriminating ability of the markers. In the case of biallelic markers, the closer MAF is to 0.5, the better [29]. In the present study, the average MAF among the 226 SNPs was 0.244, with seven SNP showing a MAF between 0.4 and 0.5 but also 33 SNP with MAF below 0.1. The minimum MAF registered was 0.023 at SNP1045_291, SNP1419_186, SNP217_190, and Vvi_3947 loci; whereas, the maximum MAF observed was 0.492 at SNP1335_204, SNP575_128, SNP663_578, and SNP855_103 loci. The average MAF was slightly lower than *V. vinifera* spp. *sativa* germplasm studied by Emanuelli et al. [7] (MAF = 0.258).

The observed number of effective alleles (*Ne*) differed from 3.928 (VVMD7) to 6.995 (VVMD5), and an average number of 4.962 effective alleles was obtained for nSSR markers, similar to the mean *Ne* value (4.658) attained by Castro et al. [4]. For SNP markers, *Ne* ranged from 1.016 (SNP625_278) to 1.999 (SNP1327_56), with an average of 1.604, which is consistent with other reports on the genetic diversity of cultivated grape varieties (Table 2; *Ne* = 1.593 in [20]; *Ne* = 1.58 in [38]).

Differences between SNPs and SSRs were also observed with respect to heterozygosity. The observed heterozygosity (*Ho*) varied between 0.785 (VVMD7 and VrZAG62) and 0.954 (VVMD5); the lowest expected heterozygosity (*He*) was detected at VVMD7 locus with 0.745 and the highest one at VVS2 locus with 0.820 (Table 2). The level of heterozygosity observed in this study (*Ho* = 0.859 and *He* = 0.790) was similar to that observed for other sets of grape varieties analysed with SSR markers (*Ho* = 0.833 and *He* = 0.767 in [53] and *Ho* = 0.833 and *He* = 0.769 in [4]). This high level of heterozygosity is in agreement with the natural breeding system of the species and could be a consequence of both natural and human selection against homozygosity in these plants [10,21,54]. Tests for Hardy-Weinberg equilibrium (HWE) revealed no significant deviations (*p* < 0.05) from HWE at the six SSR loci analysed. 

As expected, SSR loci, due to the SSR multiallelic nature and high level of polymorphism, exhibited a significantly higher heterozygosity than biallelic SNP loci. This trend was observed by Emanuelli et al. [7]. On average, SNPs displayed lower observed (*Ho* = 0.378) and expected (*He* = 0.351) heterozygosity values than SSRs (Table 2). For 196 SNP loci, no difference (*p* < 0.05) between *Ho* and *He* values was found. However, deviation from the HWE (*p* < 0.05) was observed for 13.27% of the SNP markers; for 23 SNPs (10.18%), *Ho* was significantly higher than *He*, whereas *Ho* was significantly lower than *He* in the remaining seven SNPs (3.09%).

The genotype level of polymorphism was assessed by calculating the *PIC* values for each of the six nSSR and 226 SNP loci. VVMD7 and VVMD5 markers displayed the minimum (0.736) and maximum (0.844) *PIC* values, respectively (Table 2 and Table S5). The nSSR loci were highly polymorphic and showed a mean *PIC* value of 0.773. This was in agreement with findings in other studies on Portuguese native grape genotypes (*PIC* = 0.738 in [53]; *PIC* = 0.741 in [4]). *PIC* values estimated for SNP loci, with an average of 0.280, varied between 0.006 (SNP817_209) and 0.492 (SNP853_312; Table 2). One hundred and sixty-nine SNPs displayed *PIC* values comprised between 0.2 and 0.5, and the remaining 57 showed *PIC* values below 0.2. These values indicate that the majority of SNP loci analysed had a very high discriminating capacity for grape varieties. Similar mean *PIC* values have been also reported in genetic diversity studies on *V. vinifera* varieties (*PIC* = 0.253 in [25]; *PIC* = 0.315 in [29]; *PIC* = 0.280 in [42]). *PIC* values for SNP were lower than for SSR markers due to the SNP bi-allelic nature and a maximum *PIC* value of 0.50 is usually expected for a specified SNP locus [7]. However, this can be resolved either by using a larger set of SNP markers [55] or by considering SNPs as multiallelic molecular markers [25].

The global probability of identity (PI) obtained for the six SSR set (1.0 × 10^−7^) was considerably higher than that determined for the 226 SNP set (1.4 × 10^−70^; Table 2), with the 226 SNP set having an equivalent discriminating power as a 61 SSR set. Moreover, even the 43 SNP loci (6.8 × 10^−16^) used for the varietal identification would give a similar identification power as 14 microsatellites. 

### 2.3. Chlorotype Diversity

The chlorotype (*chl*) of the 65 non-redundant genotypes was determined using ccmp3, ccmp5, and ccmp10 loci, which were considered the three most polymorphic chloroplastidial microsatellite (cpSSR) loci in grapevine [18,19].

Chlorotype diversity found in the present research work is shown in Table 3 (and Supplementary Tables S3 and S4). At least two allele variants were detected per cpSSR locus: two different size variants were found at the ccmp3 and ccmp5 loci (106 and 107 bp and 102 and 103 bp, respectively); and three different size variants at the ccmp10 locus (114, 115 and 116 bp; Table 3). The combination of alleles from these cpSSRs enabled distinguishing the main grape *chl*, designated A, B, C, and D, according to Arroyo-García et al. [19]. Chlorotype were also confirmed through SNP_NG_C_001 (C/T), SNP_NG_C_003 (C/T), and SNP_NG_D_003 (A/G) loci amplification. Using the same nomination, CCG, CTG, TTG, and CCA nucleotide combinations were found in A, B, C, and D chlorotypes, respectively (Supplementary Table S4).

*Chl* A was the most frequent (observed in 50.77% of the grape varieties), but a high percentage of *chl* D was also detected (46.15%; Table 3). *Chl* B and *chl* C were only present in the foreign varieties ‘Dodrelyabi’ and ‘Black Monukka’, respectively (Supplementary Tables S3 and S4). *Chl* A characterises the Iberian Peninsula varieties, which is referred to as a secondary centre of domestication of *V. vinifera* L. ssp. *Vinifera;* whereas, *chl* D is more commonly observed in eastern European grape varieties [13,18,44,56]. In this sense, the existence of 16 varieties and 12 new genotypes presumably autochthonous to Portugal with *chl* D (24.46 and 18.46%, respectively) could be a consequence of crosses between non-Iberian introduced varieties and Portuguese native germplasm. Chlorotypes of ‘Carrega Branco’, ‘Nevoeira’, ‘Roseira’, and the 13 new genotypes identified were determined for the first time in this work, as no previous references were found in the literature or databases used. Most of these genotypes bear *chl* D, except ‘Nevoeira’ and ‘NG013’ (both *chl A*; Supplementary Tables S3 and S4).

### 2.4. Population Structure Analysis

**Figure 3:**
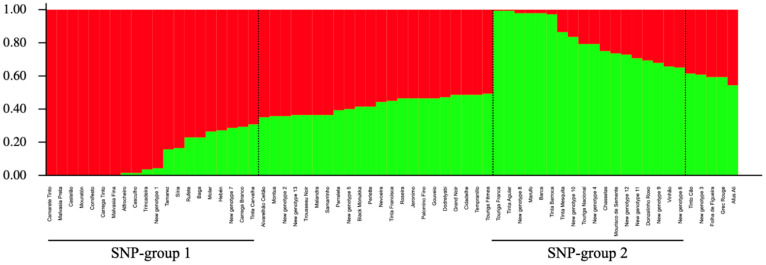
Non-redundant genotypes identified throughout “Douro” and “Trás-os-Montes” PDO regions, using 226-SNP profiles. The number of genetic groups (K = 2) was set up considering the Δ*K* criterion [57]. Every non-redundant genotype is shown as a vertical line, with colour segment lengths proportional to their inferred ancestry: genetic groups 1 and 2 are reported in red and green, respectively. Considering a critical ancestry coefficient of *q* ≥ 0.70, 20 and 18 genotypes were assigned to SNP-group 1 and SNP-group 2, respectively (with 27 admixed genotypes).

The genetic stratification in the set of 65 non-redundant grape genotypes was tested through a STRUCTURE analysis, using 226 SNP profiles. The delta *K* criterion (Δ*K*) [57] suggested K = 2 as the optimal uppermost hierarchical level of structure (Supplementary Figure S1); in this sense, genotypes divided into two major genetic groups were the best representation. Bar plot representation of the obtained structure is shown in Figure 3.

A membership coefficient (*q*-value) threshold of 0.7 for genetic group assignment was considered. Twenty (30.77%) and 18 (27.69%) genotypes were assigned to SNP-group 1 and SNP-group 2, respectively. The percentage of admixed genotypes was 41.54% (27 genotypes; Figure 3; Supplementary Table S6). SNP-group 1 is composed of Iberian varieties, including ‘Alfrocheiro’ and ‘Hebén’ that are progenitors of the majority of varieties also established in this genetic group. SNP-group 2 includes genotypes considered autochthonous to Portugal (except ‘Chasselas’), including ‘Marufo’ and ‘Touriga Nacional’ that are progenitors of the majority of varieties and five new genotypes also found in this ancestry group.

**Figure 4:**
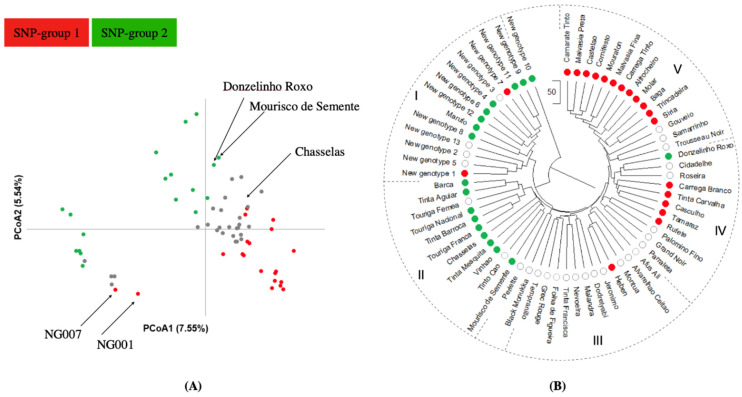
Principal coordinate analysis (**A**) and UPGMA clustering (**B**) obtained from a dissimilarity matrix calculated in GenAlEx, using 226 SNP markers from the 65 non-redundant genotypes. Genotypes assigned to ancestral genetic groups 1 and 2 are represented by red and green dots, respectively. Genotypes considered as admixtures are shown as grey (**A**) or blank (**B**) dots. In (**A**), the variance explained by the PCoA1 and PCoA2 is indicated as a percentage. In (**B**), the UPGMA clustering produced 5 major clusters (I to V).

Principal coordinate analysis (PCoA) was performed to infer the distribution of genetic relationships among structure groups as revealed by 226 SNP loci (Figure 4A). The first two PCos described 13.09% of the total variation. Ancestral groups (SNP-groups 1 and 2) were discriminated by this analysis, as both were separated along the PCo1. Admixed genotypes were generally placed in between the genotypes of each genetic group (Figure 4A). Some differences were found between PCoA (Figure 4A) and structure analysis (Figure 3). ‘NG001’ and ‘NG007’ assigned to SNP-group 1 with membership coefficients above 0.7 (0.96 and 0.71, respectively) appeared in the left part of the PCoA plot, which included genotypes from the SNP-group 2. On the contrary, ‘Donzelinho Roxo’, ‘Mourisco de Semente’, and ‘Chasselas’ assigned to SNP-group 2 with membership coefficients equal or above 0.7 (0.70, 0.74, and 0.76, respectively) appeared in the right part of the PCoA plot, which included genotypes from the SNP-group 1 (Figure 4A). These differences could be explained by the fact that the membership coefficient of all these genotypes, excluding that of ‘NG001’, is in the threshold of 0.7 attributed to genetic group assignment in population structure analysis. Nevertheless, these results highlight the risk of overinterpretation of particular data in the PCoA plot, which is based on only two coordinates explaining a limited percentage of the total variation. 

An Unweighted Pair Group Method with Arithmetic Mean (UPGMA) distance tree was constructed to investigate the genetic relationship among the 65 non-redundant genotypes from genetic distance matrices (226-SNP data; Figure 4B). Genotypes displayed different levels of similarity, ranging from 83 to 92%. Five clusters (I to V; ‘NG010’ as an outlier) were considered (Figure 4B). Clusters I and II included genotypes belonging mainly to the structure SNP-group 2, and the Clusters IV and V were mainly genotypes assigned to structure SNP-group 1. Cluster III was composed with genotypes considered admixtures (excluding ‘Hebén’ that was assigned to SNP-group 1). Hence, genotypes were clustered according to their ancestral group, but the following differences were verified: the ‘Donzelinho Roxo’ that was grouped in Cluster IV but assigned to structure SNP-group 2; and ‘NG001’ and ‘NG007’ situated in Cluster I but allocated to structure SNP-group 1 (Figure 4B). Clustering results for ‘Donzelinho Roxo’, ’NG001’, and ‘NG007’ were also supported by the PCoA (Figure 4B). Interestingly, all the new genotypes along with Marufo were included in Cluster I.

### 2.5. Pedigree Analysis

The 240 SNP-profiles of 65 non-redundant grape genotypes, including those of the 13 unique genotypes identified in the present work, were merged with those stored in the ICVV-SNP database completing a total of about 2500 genotypes, for a wide search of possible first-order kinship relationships.

Portuguese grape germplasm consisted of a very large number of varieties, and in most cases their ancestors remain largely unknown. Recently, Cunha et al. [20] reported the existence of first-degree relationships among several Portuguese varieties.

**Figure 5:**
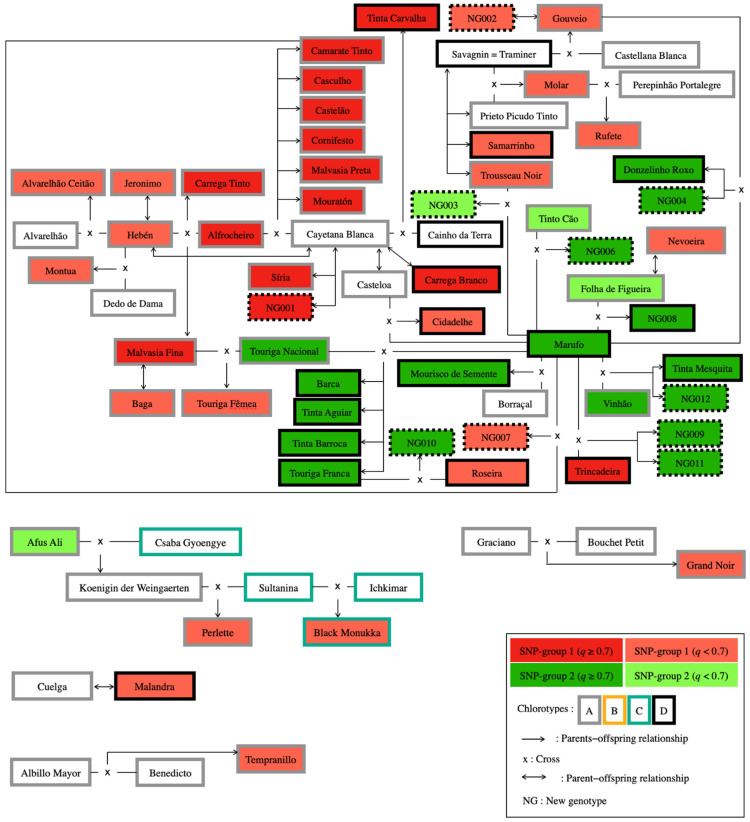
First-order genetic relationships (trios and duos) detected for grape varieties sampled in “Douro” and “Trás-os-Montes” PDO regions. These kinship relationships were obtained with the likelihood-based method implemented in CERVUS software for parentage analysis, based on 226 SNP data. Ancestral genetic groups are indicated with distinct colours (red and green) according to STRUCTURE analysis. Chlorotypes (A, B, C or D) are indicated with different colour borders, according to the inserted code. Unique genotypes in the ICVV-SNP database are shown in boxes with broken borders.

All reliable trios (both parents and offspring) and duos (parent—offspring pairs) involving genotypes analysed and the corresponding LOD values and the number of mismatching loci are presented in Table 4 and Table 5. The most relevant parentage relationships are also shown in Figure 5.

Pedigree results revealed 36 compatible trios, with high LOD values, ranging from 52.20 to 101.40, using a maximum of two mismatching loci as threshold, with the confirmation of 27 already reported trios (see Table 4 for references). The case of ‘Marufo’ and ‘Borraça’ as parents of ‘Mourisco de Semente’ was also considered reliable, despite the detection of four mismatching loci, since this trio was confirmed by Lacombe et al. [58] with 20 SSR markers. Pedigree analysis also allowed the discovery of the probable genetic origins of 9 out of 13 new genotypes (LOD values above 52; Table 4; Figure 5).

Several grape varieties have been previously reported to have an important role in the establishment of local genetic networks, such as ‘Hebén’, ‘Alfrocheiro’, and ‘Marufo’ in the Iberian Peninsula [20,59,60]. In fact, data analysis showed the significant contribution of ‘Marufo’ and ‘Alfrocheiro’ in the generation of Portuguese grapevine diversity, being involved as progenitors in 16 and 8 pedigrees, respectively.

Unlike hermaphrodite grape varieties, female progenitors, such as ‘Marufo’ (*chl* D) and ‘Hebén’ (*chl* A) need to cross-pollinate to produce descendants, a process that increases genetic diversity and increases hybrid plant vigour, which could have favoured their selection as seed donors by early farmers to ensure grape production [20].

Hence, ‘Marufo’, described as from Northeast Portugal, was found to be the mother in eight trios, which was consistent with other studies [4,20,59]. For example, pedigree data confirmed the participation of ‘Marufo’ and ‘Touriga Nacional’ (*chl* A), a variety classified by Lacerda Lobo [64] as being from “Douro” and “Beiras” wine subregions, in four known trios (Table 4; Figure 5). On the other hand, eight new descendants were also found, all bearing *chl* D as the female progenitor ‘Marufo’ and obtained from hybridisation events between the referred variety and seven different Portuguese (six) and French (one) varieties (Figure 5; Table 4). ‘NG004’, along with ‘Donzelinho Roxo’, are offspring of ‘Marufo’ and ‘Gouveio’ (*chl* A), an old variety from Douro wine region. ‘NG006’, ‘NG007’, ‘NG008’, and ‘NG003’ resulted from the cross between ‘Marufo’ and, respectively, ‘Tinto Cão’, ‘Camarate Tinto’, ‘Folha de Figueira’, and ‘Trousseau Noir’ (all male progenitors with *chl* A). Moreover, two new pedigrees were proposed as a result of crosses between ‘Marufo’ and ‘Trincadeira’ (‘NG009’ and ‘NG011’ as progenies). The participation of ‘Marufo’ and ‘Vinhão’ (*chl* A) as genitors of ‘Tinta Mesquita’ and ‘NG012’ was also observed (Table 4; Figure 5).

As previously mentioned, ’Marufo as well as ‘Tinto Cão’ and ‘Vinhão’ are late-maturing varieties. ‘Tinto Cão’ has been described in the Douro region since the XVIIl century [64]. ‘Vinhão’ is originated from North Portugal, and according to the French ampelographer Paul Truel, was introduced in Douro from the Minho region in 1790, to improve the colour of Douro wines (cited in [65]).

‘Alfrocheiro’ (*chl* A) was found to be a parent in eight trios previously described, two of them together with ‘Hebén’ (*chl* A), and six with ‘Cayetana Blanca’ (syn. ‘Sarigo’, with *chl* A) [20,59,60].

In some cases, the fact that genotypes were considered admixture (assuming a threshold *q*-value > 0.7 for group assignment) could be explained by parentage analysis. For example, both ‘Touriga Fêmea’ and ‘NG007’ were determined as admixture genotypes; ‘Touriga Fêmea’ is a progeny derived from ‘Malvasia Fina’ and ‘Touriga Nacional’ (assigned to SNP-group 1 and SNP-group 2, respectively), whereas ‘NG007’ is a reliable result of a cross between ‘Marufo’ and ‘Camarate Tinto’ (allocated to SNP-group 2 and SNP-group 1, respectively; Figure 3; Figure 5; Table 4).

Although several compatible duos were also identified, the existence of a compatible duo may not mean necessarily a parent–offspring relationship; since some are siblings or close-related varieties, they are compatible for most of the molecular markers used. In this sense, only duos more consistent (with LOD scores above 25.00 and a maximum of one mismatching loci) were considered. Five reliable duos were detected and summarised in Table 5. 

However, no reliable trios or duos within the ICVV-SNP database were found for other genotypes, mainly the Iberian ones: ‘Tamarez’, ‘Tinta Francisca’, ‘NG005’, and ‘NG013’. Since the ICVV-SNP database includes a high number of Iberian profiles and even so no parentage relationships were established, most likely progenitors of the referred genotypes are extinct or close to. Their extinction, as proposed by Cunha et al. [20], may be due to: the appearance in the 19th century of different disease-causing agents (e.g., mildews and grape phylloxera pests) that massively annihilated cultivated and wild grapevines throughout Europe; or they were minor varieties (or individual plants) lost along the evolution of viticulture due to other causes.

## 3. Materials and Methods 

### 3.1. Sampling and DNA Extraction

To analyse the ancient genetic diversity of *V. vinifera* in “Douro” and “Trás-os-Montes” PDO regions, 310 plants were sampled across 11 different old mixed variety vineyards, all predating the 1970s (Figure 1; Supplementary Table S1). The selection of plants was based on the difficulty of their morphological identification by ampelographers; the identity of 279 grape samples was unknown and had no names, whereas 31 were named mainly with local names (Supplementary Table S2).

All plants were labelled in the vineyards and young leaves were collected in several exploration trips between 2016 and 2019. Samples were kept on ice until storage at −80 °C for DNA isolation and genotyping. 

Genomic DNA was extracted according to Doyle & Doyle [66], with some modifications. Total purified DNA was detected by 1.0% (*w*/*v*) agarose gel electrophoresis containing Gel-Green^TM^ Nucleic Acid Gel Stain 0.5x (Biotium, Fremont, CA, USA) and stored at −20 °C until use. The final concentration was confirmed using a NanoDrop^®^ ND-1000 UV-Vis spectrophotometer (Thermo Fisher Scientific, Waltham, MA, USA).

### 3.2. Genotyping and Varietal Identification through SSR and SNP Markers

#### 3.2.1. SSR Markers

Samples were analysed with the set of six nuclear microsatellite (nSSR) loci recommended by OIV [41] for *Vitis* characterisation—VVS2, VVMD5, VVMD7, VVMD27, ssrVrZAG62, and ssrVrZAG79. The forward primer of each pair was fluorescently labelled with 6-FAM (VVMD5 and VVMD27), VIC (VVMD7 and ssrVrZAG62), or NED (VVS2 and ssrVrZAG79). Two multiplex PCRs (A and B) were carried out as previously described by Castro et al. [4], with 0.75 μM BSA added to each 20-μL reaction mixture. Amplifications were carried out in a TProfessional basic thermocycler (Biometra) initially set at 95 °C for 5 min., followed by 40 cycles of 94 °C/45 s, 50 °C/60 s, and 72 °C/90s, and with a final extension at 72 °C for 15 min.

The chlorotype was determined using the three chloroplastidial microsatellite (cpSSR) loci designed by Weising & Gardner [67]: ccmp3, ccmp5, and ccmp10 loci were amplified for all distinct genotypes, according to Castro et al. [68]. The forward primer of each pair was fluorescently labelled with 6-FAM (ccmp3), VIC (ccmp5), or NED (ccmp10). The PCR programme comprised an initial denaturation step at 94 °C for 3 min, followed by 30 cycles of 94 °C/45 s, 50 °C (ccmp10) or 55 °C (ccmp3 and ccmp5)/45 s, and 72 °C/60 s, and with a final extension at 72 °C for 5 min.

All PCR products were visualised by electrophoresis on 2.0% agarose gels (*w*/*v*) containing Gel-Green^TM^ Nucleic Acid Gel Stain 0.5x (Biotium). Fluorescently labelled cp and nSSR products were separated by capillary electrophoresis using the ABI PRISM^®^ 3130 automated sequencer (Applied Biosystems, Life Technologies, Foster City, CA, USA) and GeneScan^TM^ 500 LIZ^®^ (Applied Biosystems, Life Technologies, Foster City, CA, USA) as the internal lane size standard. Data produced were analysed by Peak Scanner v1.0 software (Applied Biosystems, Foster City, CA, USA). The sizes of the amplicons were scored in base pairs (bp) based on the relative migration of the internal size standard.

The varietal identification was achieved by comparing the obtained nSSR profiles with those on literature data and the *V*IVC database, with 5424 profiles [39].

#### 3.2.2. SNP Markers

DNA samples from non-redundant genetic profiles (previously identified through nSSR profiles/*V*IVC database), and samples that produced nSSR profiles that did not match with those of the *V*IVC database were also genotyped for a set of 240 SNP markers previously identified by Lijavetzky et al. [25] and Cabezas et al. [29]. Three ctSNP (SNP_NG_C_001, SNP_NG_C_003 and SNP_NG_D_003) loci were used for chlorotype determination.

The SNP genotyping was carried out as recently described in Cunha et al. [20] and Maraš et al. [38], through the Fluidigm (San Francisco, CA, USA) technology. Genotyping services were provided by the Sequencing and Genotyping Unit of the University of the Basque Country. SNP profiles obtained for the 240 SNPs were pairwise compared with those of the ICVV-SNP database for varietal identification.

### 3.3. Data Analyses

Data obtained with the nSSR loci were scored based on the molecular size (in bp) of alleles. For SNP data, numerical values were assigned to each nucleotide (missing data = 0; A = 1, C = 2, G = 3, T = 4).

Non-redundant grapevine genotypes with genetic profiles for 6 nSSR and 48 SNP loci were used for grape variety identification. Genetic profiles for 240 SNP loci were used for population structure and genetic diversity analyses.

#### 3.3.1. Genetic Diversity Analysis

To compare SSR and SNP results, genetic parameters of polymorphism, such as the average number of different alleles per locus (*Na*), the average number of effective alleles (*Ne*), observed heterozygosity (*Ho*) and gene diversity or expected heterozygosity (*He*) were calculated through the GenAlEx software (version 6.5) [69]. They were determined from single-locus values. The same software was also used to test for deviation from the Hardy-Weinberg equilibrium (HWE) across all loci for each population.

Polymorphism information content (PIC) [70] of each nSSR marker was determined using an online tool [71]. For SNP markers, *PIC* was calculated as follows: *PIC* = *He* − (2 × MAF^2^ × (1-MAF)^2^), where MAF was the minor allele frequency.

#### 3.3.2. Population Structure Analysis

Structure analyses were performed using the STRUCTURE software (version 2.3.4) [72,73,74,75], using 240-SNP data. This model was carried out to evaluate the number of inferred genetic population clusters (K) and to assign individuals to their likely population of origin, using no prior information. An initial burn-in of 20,000 steps was used to minimise the effect of the starting configuration, followed by 100,000 Markov Chain Monte Carlo (MCMC) steps, as recommended by Falush et al. [74] and Ghaffari et al. [21], under the admixture model and independent allele frequencies. Ten replicate runs per K value were set up, with K ranging from 1 to 10. To identify the number of K clusters explaining the observed genetic structure, the log-probability of the data (LnP(D)) in STRUCTURE output as well as the delta K values were obtained, using the online available program STRUCTURE HARVESTER (web version 0.6.94) [76], and based on the Evanno et al. [57] method. Samples were assigned probabilistically to genetic groups according to their membership coefficient (*q*-value). 

To assess the relationship among the non-redundant genotypes, the pairwise genetic distance matrix was computed based on SNP data, through the ‘Genetic Distance’ procedure in the GenAlEx software (version 6.5) [69], for subsequent analyses. Principal coordinate analyses (PCoA) were performed using the same software for SNP distance matrices, conducted on individual multilocus genotypes and with covariance standardised. The clustering was inferred using the Unweighted Pair Group Method with Arithmetic Mean (UPGMA) [77]. The optimal circular trees obtained for SNP markers were plotted using MEGA X software [78]. A tree was drawn to scale, with branch lengths in the same units as those of the evolutionary distances used to infer the phenetic tree.

#### 3.3.3. Pedigree Analysis

Data from the 240 SNP set were also used to identify possible first-order kinship relationships—trios (mother–father–offspring) and duos (possible parent–offspring pairs)—among the non-redundant grape genotypes in the study, since their SNP profiles merged with those of the ICVV-SNP database. The likelihood-based method in the CERVUS software was used (version 3.0) [79]. The likelihood of each detected trio and duo detected was determined based on the natural logarithm of the overall likelihood ratio—logarithm-of-odds (LOD) score—and a maximum number of mismatching loci of 1 or 4 SNPs for duos and trios, respectively. Where possible, chlorotypes were used to infer which of the putative parents was the maternal progenitor in each trio [13,19].

## 4. Conclusions

SSR and SNP analyses were very useful in the identification and characterisation of the plants analysed and overcame the ampelography difficulties in grapevine prospections to contribute to the ultimate goal of conserving grape varietal legacy in Northeast Portugal. Further morphological, agronomical, and oenological analyses are being undertaken to complement the molecular data reported in this study.

In the present work, 280 plants in the “Douro” and “Trás-os-Montes” PDO regions were identified and grouped into 52 different grape varieties. Thirteen additional unique genotypes were also detected from the study of other 30 vines, which were clustered, along with cv. Marufo (mother of the majority of these new genotypes), in an exclusive independent cluster, accordingly to UPGMA data. Some of these 13 new genotypes could be considered minor neglected Portuguese grape varieties, while others, found only in plants belonging to a single local vine grower still using traditional techniques for grapevine propagation, are probably individual grapevines that emerged as seedlings and can be the initial steps of the establishing of local grape varieties as it occurred in the past. Altogether, their discovery highlights once more the huge patrimony of the Portuguese grape germplasm. 

The aforementioned observations emphasise the constant demand for prospection and identification of old grape material in traditional vineyards, to enlarge the knowledge about the still existing varietal diversity for its conservation, characterisation, and eventual exploitation. The broader the knowledge, the greater the chances to overcome biotic and abiotic stresses affecting the vineyards today.

## Figures and Tables

**Table 1:** List of the 65 *Vitis vinifera* L. genotypes identified and respective sample origin, genotype code numbers in international databases, synonymies, berry colour, and grape utilisation.

Sample Origin ^1^ (Number of Samples Analysed)	Cultivar Prime Name ^2^	*V*IVC Variety No.	ICVV-SNP Genotype No.	Synonymies in Portugal	Colour of Berry Skin ^3^	Grape Use
QC (1)	Afus Ali	122	GEN_DNA_2036		B	Wine/Table
J (1)	Alfrocheiro (D) *	277	GEN_DNA_2173	Tinta Bastardinha	N	Wine
QSI (1)	Alvarelhão Ceitão (D) *	368	GEN_DNA_2837		R	Wine
Qs (1)	Baga (D,T) *	885	GEN_DNA_1267		N	Wine
QC (1), M (1)	Barca (D) *	17359	GEN_DNA_3010		N	Wine
QSI (1), Vs (1)	Black Monukka	17452	GEN_DNA_2127		N	Table
V (2), Qs (1), QC (6), J (1)	Camarate Tinto *	2018	GEN_DNA_0634		N	Wine
Ag (2), Sd (9)	Carrega Branco (D,T) *	2124	GEN_DNA_2199		B	Wine
R (1)	Carrega Tinto (D) *	2125	GEN_DNA_1270	Tinta Grossa	N	Wine
Sd (13), Qs (1), QC (9)	Casculho (D) *	14149	GEN_DNA_2976		N	Wine
V (1), QC (1)	Castelão (D,T) *	2324	GEN_DNA_1168		N	Wine/Table
Vs (1), QSI (1)	Chasselas (D)	2473	GEN_DNA_2055		B	Wine/Table
Qs (1)	Cidadelhe (D) *	12476	GEN_DNA_2997		N	Wine
Sd (1), QC (1), J (1)	Cornifesto (D,T) *	2846	GEN_DNA_1229		N	Wine
QSI (4)	Dodrelyabi	3616	GEN_DNA_0984		N	Wine/Table
Vs (2)	Donzelinho Roxo *	17677	GEN_DNA_2964		R	Wine
Ag (1), V (1)	Folha de Figueira (D) *	14142	GEN_DNA_3002	Dona Branca	B	Wine/Table
Ag (2), Sd (2)	Gouveio (D,T) *	12953	GEN_DNA_1133		B	Wine
Qs (1)	Grand Noir (D)	5012	GEN_DNA_1110		N	Wine
J (1)	Grec Rouge	4962	GEN_DNA_1212	Rabigato Franco	R	Wine/Table
QSI (2)	Hebén (D)	5335	GEN_DNA_1258	Mourisco Branco	B	Wine/Table
Vs (1)	Jeronimo	5692	GEN_DNA_2236		N	Wine/Table
M (3), J (2)	Malandra (D) *	12487	GEN_DNA_2967		N	Wine
QC (1)	Malvasia Fina (D,T) *	715	GEN_DNA_2245		B	Wine
QC (11)	Malvasia Preta (D,T) *	15647	GEN_DNA_2347		N	Wine
Ag (1), Vs (4), QSI (1), Sd (1), V (2), Qs (1), QC (3)	Marufo (D,T) *	8086	GEN_DNA_1205		N	Wine/Table
Ag (1)	Molar *	15678	GEN_DNA_2128	Tinta Negra	N	Wine/Table
Vs (2)	Montua (D)	2520	GEN_DNA_0621	Diagalves	B	Wine/Table
Ag (1), Vs (3), Sd (12), Qs (1)	Mouratón (T)	8082	GEN_DNA_2201	Tinta Gorda	N	Wine
QC (7)	Mourisco de Semente (D) *	12471	GEN_DNA_2999		N	Wine
QC (6)	Nevoeira (D) *	8504	GEN_DNA_3008		N	Wine
QSI (6)	Palomino Fino (D)	8888	GEN_DNA_1063	Malvasia Rei	B	Wine/Table
QC (1)	Parraleta (D)	8951	GEN_DNA_1003	Tinta Caiada	N	Wine
Sd (2)	Perlette	9168	GEN_DNA_0148		B	Table/Raisin
Qs (6), QC (10)	Roseira (D) *	12497	GEN_DNA_2971		N	Wine
Qs (1)	Rufete (D,T) *	10331	GEN_DNA_2106	Tinta Pinheira	N	Wine
Vs (1)	Samarrinho (D,T) *	15684	GEN_DNA_0856	Budelho	B	Wine
Vs (2), QSI (7), Sd (8)	Síria (D,T) *	2742	GEN_DNA_1154	Roupeiro, Códega	B	Wine/Table
R (1)	Tamarez (D) *	12231	GEN_DNA_2224	Molinha	B	Wine
Vs (1), Sd (2), V (8), QC (2)	Tempranillo (D,T)	12350	GEN_DNA_1316	Aragonez, Tinta Roriz	N	Wine/Table
V (1), C (1)	Tinta Aguiar (D) *	12459	GEN_DNA_2968		N	Wine
Sd (1), QC (1)	Tinta Barroca (D,T) *	12462	GEN_DNA_1167		N	Wine
Vs (2), V (2), QC (2), M (1)	Tinta Carvalha (D,T) *	12467	GEN_DNA_1123		N	Wine
V (4), Qs (1), QC (2)	Tinta Francisca (D) *	15686	GEN_DNA_2348		N	Wine
C (1)	Tinta Mesquita (D) *	12489	GEN_DNA_3215		N	Wine
Vs (1), Sd (1), QC (2)	Tinto Cão (D,T) *	12500	GEN_DNA_0651		N	Wine
QC (4)	Touriga Fêmea (D) *	12592	GEN_DNA_2969	Touriga Brasileira	N	Wine
Sd (1), QC (5), M (1)	Touriga Franca (D,T) *	12593	GEN_DNA_0493		N	Wine
V (2), Qs (1), QC (3)	Touriga Nacional (D,T) *	12594	GEN_DNA_0760		N	Wine
Vs (2), QSI (9), Sd (4), V (8), QC (2), J (3)	Trincadeira (D,T) *	15685	GEN_DNA_1239	Tinta Amarela, Trincadeira Preta	N	Wine
Vs (1), QSI (3), Sd (4), Qs (1)	Trousseau Noir (D,T)	12668	GEN_DNA_2156	Bastardo	N	Wine
Qs (1), QC (2)	Vinhão (D,T) *	13100	GEN_DNA_2240	Sousão	N	Wine
Ag (3)	NG001		GEN_DNA_4342		B	Wine
Ag (1)	NG002		GEN_DNA_4343		B	Wine
Ag (1)	NG003		GEN_DNA_4344		N	Wine
Ag (1)	NG004		GEN_DNA_4345		R	Wine
Sd (1)	NG005		GEN_DNA_4346		B	Wine
QC (4)	NG006		GEN_DNA_4347		N	Wine
QC (4)	NG007		GEN_DNA_4335		N	Wine
QC (4)	NG008		GEN_DNA_4336		N	Wine
QC (4)	NG009		GEN_DNA_4337		N	Wine
QC (1)	NG010		GEN_DNA_4348		N	Wine
Qs (1)	NG011		GEN_DNA_4349		B	Wine
C (1)	NG012		GEN_DNA_4350		N	Wine
J (7)	NG013		GEN_DNA_4338		N	Wine

^1^ Ag—Aguieiras; C—Quinta do Cruzeiro; J—Quinta do Junco; M—Quinta dos Muros; QC—Quinta das Carvalhas; Qs—Quinta do Seixo; QSI—Quinta de Santa Isabel; Sd—Sendim; V—Quinta dos Lagares; Vs—Vassal; R—Quinta da Roêda. ^2^ Varieties presumably autochthonous to Portugal were marked by an asterisk and those authorised in ‘Douro’ and/or ‘Trás-os-Montes’ PDO regions with (D) and/or (T), respectively. ^3^ N—Noir; R—Rouge; B—Blanc.

**Table 2:** Genetic parameters estimated for nSSR and SNP profiles from the 65 grape genotypes studied.

	6 nSSR Markers			226 SNP Markers		
	Minimum	Maximum	Mean ± SE	Minimum	Maximum	Mean ± SE
*Na*	7 (VVMD7)	12 (VVS2)	9.500 ± 0.671	-	-	2.000
*Ne*	3.928 (VVMD7)	6.995 (VVMD5)	4.962 ± 0.480	1.016 (SNP625_278)	1.999 (SNP1327_56)	1.604 ± 0.020
*Ho*	0.785 (VVMD7; VrZAG62)	0.954 (VVMD5)	0.859 ± 0.027	0.016 (SNP625_278)	0.714 (SNP251_159)	0.378 ± 0.011
*He*	0.745 (VVMD7)	0.820 (VVS2)	0.790 ± 0.018	0.016 (SNP625_278)	0.499 (SNP895_382; VMFT_595; Vvi_1187; Vvi_10992)	0.351 ± 0.009
*PIC*	0.736 (VVMD7)	0.844 (VVMD5)	0.773 ± 0.015	0.006 (SNP817_209)	0.492 (SNP853_312)	0.280 ± 0.009
*PI*	0.037 (VVMD5))	0.095 (VrZAG79)	0.072 ± 0.022	0.375 (SNP1495_148)	0.969 (SNP625_278)	0.510 ± 0.152

*Na*—Average number of different alleles per locus; *Ne*—number of effective alleles; *Ho*—observed heterozygosity; *He*—expected heterozygosity; *PIC*—Polymorphism information content; *PI*—Probability of identity.

**Table 3:** List of allele sizes (in bp, x represents presence of the allele) and their frequency of each chloroplastidial microsatellite loci analysed. The corresponding chlorotype and their frequency observed in the 52 grape varieties and 13 new genotypes are also shown.

Chlorotype	Loci	Ccmp3		Ccmp5		Ccmp10			Frequency (%)
	Allele Sizes (bp)	105	106	103	102	110	111	112	
A	Combination of ccmp alleles	x		x		x			50.77
B		x		x			x		1.54
C		x		x				x	1.54
D			x		x		x		46.15
	Frequency (%)	53.85	46.15	53.85	46.15	50.77	47.69	1.54	

**Table 4:** List of trios (parents–offspring) identified in this study using 226 SNP data. Genotypes for which a pedigree has been confirmed with molecular markers for the first time are highlighted in bold.

Offspring				Parent 1				Parent 2				Trio Loci Compared	M ^2^	Trio LOD Score	References
ICVV-SNP Genotype Number	*V*IVC Number	*Chl* ^1^	Variety/New Genotype Name	ICVV-SNP Genotype Number	*V*IVC Number	*Chl* ^1^	Variety Name	ICVV-SNP Genotype Number	*V*IVC Number	*Chl* ^1^	Variety Name				
2837	368	A	Alvarelhão Ceitão	1258	5335	A	Hebén	815	1650	A	Alvarelhão	225	0	77.42	[20]
3010	17359	D	Barca	1205	8086	D	Marufo	760	12594	A	Touriga Nacional	216	0	78.02	[20,58]
2127	17452	C	Black Monukka	2126	12051	C	Sultanina	463	5477	C	Ichkimar	228	2	81.71	[58]
634	2018	A	Camarate Tinto	1089	5648	A	Cayetana Blanca	2173	277	A	Alfrocheiro	230	0	83.17	[20,58,59,60]
1270	2125	A	Carrega Tinto	1258	5335	A	Hebén	2173	277	A	Alfrocheiro	226	0	81.29	[20,59,60]
2976	14149	A	Casculho	1089	5648	A	Cayetana Blanca	2173	277	A	Alfrocheiro	218	1	74.67	[20,60]
1168	2324	A	Castelão	1089	5648	A	Cayetana Blanca	2173	277	A	Alfrocheiro	236	0	94.09	[20,58,59,60]
2997	12476	D	Cidadelhe	1205	8086	D	Marufo	2960	23126	A	Casteloa	217	1	66.16	[20]
1229	2846	A	Cornifesto	1089	5648	A	Cayetana Blanca	2173	277	A	Alfrocheiro	230	0	75.51	[20,58,59,60]
2964	17677	D	Donzelinho Roxo	1205	8086	D	Marufo	1133	12953	A	Gouveio	220	1	68.57	[20]
1133	12953	A	Gouveio	2397	40016	A	Castellana Blanca	2099	17636	D	Savagnin = Traminer	234	0	65.72	[20,58]
1110	5012	A	Grand Noir	1321	4935	A	Graciano	2204	1619	A	Bouschet Petit	232	0	79.31	[58]
2245	715	A	Malvasia Fina	1258	5335	A	Hebén	2173	277	A	Alfrocheiro	238	1	77.80	[20,58,59,60]
2347	15647	A	Malvasia Preta	1089	5648	A	Cayetana Blanca	2173	277	A	Alfrocheiro	236	0	90.48	[20,58,59,60]
2128	15678	A	Molar	939	9694	A	Prieto Picudo Tinto	2099	17636	D	Savagnin = Traminer	233	1	56.90	[20]
621	2520	A	Montua	1258	5335	A	Hebén	2306	14842	C	Dedo de Dama	238	0	101.41	[20,58,59]
2201	8082	A	Mouratón	1089	5648	A	Cayetana Blanca	2158	277	A	Alfrocheiro	236	1	83.62	[20,59,60]
2999	12471	D	Mourisco de Semente	1205	8086	D	Marufo	896	1564	A	Borraçal	220	4	54.53	[20,58]
148	9168	A	Perlette	2035	6350	A	Koenigin der Weingaerten	2126	12051	C	Sultanina	223	1	78.13	[59,61,62]
2106	10331	A	Rufete	2128	15678	A	Molar	3960	21437	A	Perepinhão Portalegre	237	0	77.41	[20,58]
1316	12350	A	Tempranillo	2410	1131	A	Benedicto	2228	12581	A	Albillo Mayor	234	0	100.00	[20,63]
2968	12459	D	Tinta Aguiar	1205	8086	D	Marufo	760	12594	A	Touriga Nacional	219	0	68.59	[20]
1167	12462	D	Tinta Barroca	1205	8086	D	Marufo	760	12594	A	Touriga Nacional	236	0	75.72	[20,58]
1123	12467	D	Tinta Carvalha	3088	26692	D	Cainho da Terra	1089	5648	A	Cayetana Blanca	222	1	59.15	[20]
3215	12489	D	Tinta Mesquita	1205	8086	D	Marufo	2240	13100	A	Vinhão	225	1	61.44	[20]
2969	12592	A	Touriga Fêmea	2245	715	A	Malvasia Fina	760	12594	A	Touriga Nacional	215	1	64.23	[20,58]
493	12593	D	Touriga Franca	1205	8086	D	Marufo	760	12594	A	Touriga Nacional	236	1	69.51	[4,20]
4344		D	NG003	1205	8086	D	Marufo	2156	12668	A	Trousseau Noir	213	2	52.20	this study
4345		D	NG004	1205	8086	D	Marufo	1133	12953	A	Gouveio	210	1	58.71	this study
4347		D	NG006	1205	8086	D	Marufo	651	12500	A	Tinto Cão	212	2	58.39	this study
4335		D	NG007	1205	8086	D	Marufo	634	2018	A	Camarate Tinto	210	0	64.63	this study
4336		D	NG008	1205	8086	D	Marufo	3002	14142	A	Folha de Figueira	213	2	76.00	this study
4337		D	NG009	1205	8086	D	Marufo	1239	15685	D	Trincadeira	213	1	58.54	this study
4348		D	NG010	2971	12497	D	Roseira	493	12593	D	Touriga Franca	162	0	58.91	this study
4349		D	NG011	1205	8086	D	Marufo	1239	15685	D	Trincadeira	213	1	67.72	this study
4350		D	NG012	1205	8086	D	Marufo	2240	13100	A	Vinhão	212	1	65.43	this study

^1^ Chl—Chlorotype. ^2^ M—Trio loci mismatching.

**Table 5:** Possible duos (parent–offspring relationship) found in a parentage analyses from 5 genotypes.

Offspring				Parent 1				Pair Loci Compared	Pair Loci Mismatching	Pair LOD Score
ICVV-SNP Genotype Number	Chlorotype	Genotype Name	Loci Typed	ICVV-SNP Genotype Number	Chlorotype	Genotype Name	Loci Typed			
4342	D	NG001	213	1089	A	Cayetana Blanca	235	210	0	33.50
4343	D	NG002	212	1133	A	Gouveio	236	210	1	30.71
2199	D	Carrega Branco	218	1089	A	Cayetana Blanca		215	0	30.77
2967	D	Malandra	214	2450	A	Cuelga		210	0	28.40
3008	A	Nevoeira	230	3002	A	Folha de Figueira		221	0	25.86

## Data Availability

Data is contained within the article and supplementary file.

## Supplementary Materials

The following are available online at https://www.mdpi.com/article/10.3390/plants10122755/s1, Table S1. List of *Vitis vinifera* L. cultivars (52) and new genotypes (13) identified and corresponding sample codes; Table S2. List of 31 grape samples with local names and SSR and SNP identifications according to the legal cultivar name in Portugal; Table S3. List of the 65 grape genotypes detected at 6 nSSR and 3 cpSSR loci analysed; Table S4. List of the 65 non-redundant grape genotypes detected at 46 polymorphic SNP loci; Table S5. Genetic parameters, allele sizes, and frequencies over 6 microsatellite loci in the 65 non-redundant genotypes analysed in this study; Table S6. Structure results at K = 2 based on 226 SNP markers. Genotypes with membership coefficients (*q*-values) below the threshold of 0.7 for genetic group assignment were admixed; Figure S1. Delta K plots obtained from STRUCTURE HARVESTER to set the most likely number of genetic groups within the 65 non-redundant grape population identified in the present study, based on 226-SNP data.
