# Supplementary material for: Grapevine Diversity and Genetic Relationships in Northeast Portugal Old Vineyards
Source: Plants (Basel). 2021 Dec 14;10(12):2755. doi: 10.3390/plants10122755 (PMC8705298; doi:10.3390/plants10122755)
Supplement: Supplementary file 1 [file plants-10-02755-s001.zip › plants-1466653-supplementary.pdf]

plants

MDPI

Article

Grapevine Diversity and Genetic Relationships in Northeast Portugal Old Vineyards

Diana Augusto [1], Javier Ibáñez [2], Ana Lúcia Pinto-Sintra [3], Virgílio Falco [4], Fernanda Leal [3], José Miguel Martínez-Zapater [2], Ana Alexandra Oliveira [3] and Isaura Castro [3,*]

Supplementary Materials: The following are available online at www.mdpi.com/xxx/s1, **Table S1.** List of *Vitis vinifera* L. cultivars (52) and new genotypes (13) identified and corresponding accession codes; **Table S2.** List of 31 grape samples with local names and SSR and SNP identifications according to the legal cultivar name in Portugal; **Table S3.** List of the 65 grape genotypes detected at 6 nSSR and 3 cpSSR loci analysed; **Table S4.** List of the 65 non-redundant grape genotypes detected at 46 polymorphic SNP loci; **Table S5.** Genetic parameters, and allele sizes and frequencies over 6 microsatellite loci in the 65 non-redundant genotypes analysed in this study; **Table S6.** Structure results at K = 2 based on 226 SNP markers. Genotypes with membership coefficients (*q* - values) below the threshold of 0.7 for genetic group assignment were admixed; **Figure S1.** Delta K plots obtained from STRUCTURE HARVESTER to set the most likely number of genetic groups within the 65 non-redundant grape population identified in the present study, based on 226 SNP data.

Table S1. List of *Vitis vinifera* L. cultivars (52) and new genotypes (13) identified and corresponding samples.

Cultivar prime name	Samples[1]
**Afus Ali**	QC7.59
**Alfrocheiro**	J38Ed
**Alvarelhão Ceitão**	QSI19
**Baga**	Qs9
**Barca**	QC4.21, M66Ed
**Black Monukka**	QSI8, Vs17
**Camarate Tinto**	V1, V28, Qs21, QC4.51, QC5.37, QC7.43, QC8.33, QC9.41, QC10.10, J68Ed
**Carrega Branco**	Ag10, Ag13, Sd6, Sd7, Sd14, Sd21, Sd23, Sd31, Sd32, Sd33, Sd56
**Carrega Tinto**	R59Ed
**Casculho**	Sd2, Sd3, Sd4, Sd9, Sd13, Sd24, Sd30, Sd35, Sd43, Sd46, Sd49, Sd52, Sd53, Qs14, QC4.28, QC4.47, QC6.69, QC7.20, QC7.41, QC8.06, QC8.31, QC8.39, QC10.58
**Castelão**	V33, QC9.27
**Chasselas**	Vs20, QSI2
**Cidadelhe**	Qs4
**Cornifesto**	Sd20, QC8.07, J75Ed
**Dodrelyabi**	QSI5, QSI7, QSI11, QSI22
**Donzelinho Roxo**	Vs21, Vs22
**Folha de Figueira**	Ag6, V10
**Gouveio**	Ag2, Ag12, Sd47, Sd63
**Grand Noir**	Qs18
**Grec Rouge**	J17Ed
**Hebén**	QSI3, QSI10
**Jeronimo**	Vs23
**Malandra**	M61Ed, M89, M90, J61Ed, J37Ed
**Malvasia Fina**	QC4.33
**Malvasia Preta**	QC4.40, QC5.14, QC5.41, QC6.04, QC6.05, QC6.25, QC7.33, QC8.37, QC8.52, QC8.62, QC9.53
**Marufo**	Ag11, Vs12, Vs14, Vs15, Vs19, QSI16, Sd58, V20, V32, Qs13, QC4.13, QC6.12, QC8.01
**Molar**	Ag1
**Montua**	Vs2, Vs4
**Mouratón**	Ag7, Vs5, Vs7, Vs10, Sd15, Sd17, Sd18, Sd28, Sd36, Sd38, Sd39, Sd41, Sd42, Sd48, Sd54, Sd55, Qs10
**Mourisco de Semente**	QC5.36, QC5.38, QC5.42, QC6.37, QC6.38, QC7.34, QC8.35
**Nevoeira**	QC4.17, QC4.53, QC5.07, QC6.36, QC6.49, QC9.28
**Palomino Fino**	QSI1, QSI12, QSI13, QSI15, QSI24, QSI28
**Parraleta**	QC6.50
**Perlette**	Sd25, Sd27

Table S1. (cont.)

**Cultivar prime name**	**Accession Code**[1]
**Roseira**	Qs1, Qs2, Qs5, Qs8, Qs11, Qs20, QC6.45, QC6.28, QC6.56, QC8.09, QC8.14, QC8.40, QC8.41, QC8.53, QC8.54, QC9.56
**Rufete**	Qs19
**Samarrinho**	Vs9
**Síria**	Vs11, Vs18, QSI6, QSI18, QSI20, QSI23, QSI29, QSI33, QSI34, Sd5, Sd10, Sd19, Sd22, Sd29, Sd34, Sd37, Sd61
**Tamarez**	R93
**Tempranillo**	Vs24, Sd44, Sd45, V4, V6, V8, V9, V14, V15, V17, V39, QC5.11, QC6.67
**Tinta Aguiar**	V44, C22Ed
**Tinta Barroca**	Sd57, QC5.21
**Tinta Carvalha**	Vs3, Vs8, V34, V47, QC6.29, QC8.15, M5.3
**Tinta Francisca**	V12, V19, V29, V50, Qs15, QC4.50, QC10.56
**Tinta Mesquita**	C24Ed
**Tinto Cão**	Vs16, Sd50, QC9.01, QC10.68
**Touriga Fêmea**	QC4.38, QC4.45, QC4.55, QC5.19
**Touriga Franca**	Sd12, QC4.30, QC5.46, QC7.09, QC9.45, QC9.57, M5.1
**Touriga Nacional**	V7, V23, Qs6, QC4.64, QC9.13, QC9.42
**Trincadeira**	Vs13, Vs27, QSI4, QSI17, QSI21, QSI25, QSI26, QSI27, QSI30, QSI31, QSI35, Sd8, Sd11, Sd51, Sd62, V5, V13, V22, V27, V30, V38, V40, V46, QC8.50, QC9.08, J36Ed, J40Ed, J92
**Trousseau Noir**	Vs25, Vs26, QSI9, QSI14, QSI32, Sd1, Sd16, Sd59, Sd60, Qs7
**Vinhão**	Qs12, QC4.62, QC8.65

**New genotypes**	**Accession Code**[1]
**NG001**	Ag3, Ag5, Vs6
**NG002**	Ag8
**NG003**	Ag9
**NG004**	Ag14
**NG005**	Sd40
**NG006**	QC4.20
**NG007**	QC4.25, QC6.09, QC6.11, QC7.23
**NG008**	QC5.17, QC7.05, QC7.06, QC8.25
**NG009**	QC5.35, QC6.66, QC8.29, QC8.47
**NG010**	QC10.42
**NG011**	Qs17
**NG012**	C25Ed
**NG013**	J18Ed, J39Ed, J42Ed, J43Ed, J44Ed, J84Ed, J91

[1] Ag - Aguieiras; C - Quinta do Cruzeiro; J - Quinta do Junco; M - Quinta dos Muros; QC - Quinta das Carvalhas; Qs - Quinta do Seixo; QSI - Quinta de Santa Isabel; Sd - Sendim; V - Quinta dos Lagares; Vs - Vassal; R - Quinta da Roêda.

Table S2. List of 31 grape samples with local names and SSR and SNP identifications according to the legal cultivar name in Portugal.

Sample code [1]	Local names	**Recommended *VIVC* name** or **provisional name** (ICVV-SNP genotype)
Ag2	Verdelheira / Verdelho	Gouveio
Ag6	Loulela / Folgazona	Folha de Figueira
Ag7	Mourisco	Mouratón
Ag8	Lázaro / Raposeira	Lázaro (NG002; GEN_DNA_4343)
Ag14	Rosada	Rosada (NG004; GEN_DNA_4347)
Vs13	Tinta Amarela Antiga	Trincadeira
Vs27	Tinta Amarela Antiga	Trincadeira
Sd2	Tinta Grossa	Casculho
Sd5	Polita	Síria
Sd12	Tinta Garcia / João Garcia	Touriga Franca
Qs1	Roseira	Roseira
Qs2	Mourisco de Semente	Roseira
Qs4	Preto Martinho	Cidadelhe
Qs5	Mesquita	Roseira
Qs6	Touriga Fêmea	Touriga Nacional
Qs7	Bastardo	Trousseau Noir
Qs8	Tinta Carvalha	Roseira
Qs9	Tinta Bairrada	Baga
Qs10	Bastardinho	Mouratón
Qs11	Intorzeira	Roseira
Qs12	Sousão	Vinhão
Qs13	Barca	Marufo
Qs14	Casculho	Casculho
Qs15	Tinta Francisca	Tinta Francisca
Qs17	Mourisco	Mourisco Falso (NG011; GEN_DNA_4349)
Qs18	Grand Noir	Grand Noir
Qs19	Rufete	Rufete
Qs20	Cornifesto	Roseira
Qs21	Moreto	Camarate Tinto
J17Ed	Rabigato Francês	Grec Rouge
C24Ed	Tinta do Bragão	Tinta Mesquita
C25Ed	Mourisco de Semente	Mourisco de Semente Falso (NG012; GEN_DNA_4350)
M5.1	Tinta Malandra	Touriga Franca

[1] Ag - Aguieiras; C - Quinta do Cruzeiro; J - Quinta do Junco; M - Quinta dos Muros; Qs - Quinta do Seixo; Sd - Sendim; Vs - Vassal.

Table S3. List of the 65 grape genotypes detected at 6 nSSR and chlorotypes obtained from 3 cpSSR loci analysed.

Genotype	nSSR sizes (bp)[1]												Chloro-types[2]	References
	VVS2		VVMD5		VVMD7		VVMD27		VrZAG62		VrZAG79			
**Afus Ali**	132	134	222	228	239	249	181	181	189	191	241	249	A	[1]
**Alfrocheiro**	142	150	222	234	253	257	175	185	191	203	249	249	A	[2]
**Alvarelhão Ceitão**	142	150	222	236	239	243	185	191	191	191	249	255	A	[3]
**Baga**	142	**154**	228	236	239	239	175	185	191	207	245	249	A	[4]
**Barca**	142	150	222	228	239	239	179	185	191	191	243	255	D	[4]
**Black Monukka**	140	150	230	236	253	253	177	191	191	191	245	255	C	[1]
**Camarate Tinto**	144	150	232	234	243	253	177	185	191	203	245	249	A	[2]
**Carrega Branco**	136	150	232	234	243	257	177	185	191	197	243	245	D	this study
**Carrega Tinto**	142	150	230	234	239	253	175	191	191	203	249	255	A	[1,2]
**Casculho**	144	150	232	234	249	253	177	185	203	207	245	249	A	[5]
**Castelão**	142	144	232	234	243	257	175	177	191	191	245	249	A	[2]
**Chasselas**	132	142	224	232	239	247	181	185	197	207	249	257	D	[4]
**Cidadelhe**	142	142	228	230	243	253	177	191	195	203	245	255	D	[1]
**Cornifesto**	142	144	230	234	249	253	177	185	203	207	245	249	A	[2]
**Dodrelyabi**	132	134	222	232	247	**255**	175	181	207	207	249	249	B	[1]
**Donzelinho Roxo**	142	150	224	234	239	243	185	191	189	195	249	255	D	[5]
**Folha de Figueira**	142	156	232	236	239	239	175	181	189	189	249	249	A	[1]
**Gouveio**	150	156	222	234	239	243	181	185	189	191	249	249	A	[2,4]
**Grand Noir**	**138**	150	222	230	239	243	177	179	191	191	241	257	A	[1]
**Grec Rouge**	132	142	224	232	247	253	181	181	191	197	**237**	249	A	[1]
**Hebén**	142	144	230	236	239	243	177	191	191	191	245	255	A	[1]
**Jeronimo**	134	142	230	234	243	243	191	191	191	191	255	255	A	[1]
**Malandra**	142	150	222	236	239	253	175	177	191	203	249	259	D	[1]
**Malvasia Fina**	142	144	222	236	239	257	175	191	191	191	245	249	A	[2,4]
**Malvasia Preta**	136	150	222	232	249	253	177	185	203	207	245	249	A	[1]
**Marufo**	142	144	224	228	239	243	179	191	191	195	245	255	D	[2,4]
**Molar**	132	150	222	234	239	257	177	185	191	197	243	259	A	[1]
**Montua**	142	150	230	234	243	253	177	181	191	191	245	255	A	[2,3]
**Mouratón**	136	150	230	234	249	257	177	185	191	207	245	249	A	[1]
**Mourisco de Semente**	134	142	228	234	239	239	177	191	191	197	245	255	D	[1]
**Nevoeira**	150	156	228	232	239	257	175	185	189	197	245	245	A	this study
**Palomino Fino**	132	144	224	236	239	249	181	191	191	197	249	255	D	[2]

Table S3. (cont.)

Genotype	nSSR sizes (bp)[1]												Chloro-types[2]	References
	VVS2		VVMD5		VVMD7		VVMD27		VrZAG62		VrZAG79			
**Parraleta**	132	132	218	234	239	239	175	185	189	191	249	259	A	[2]
**Perlette**	132	144	230	232	247	253	175	177	191	207	245	**253**	A	[1]
**Roseira**	142	142	222	228	239	253	177	191	191	203	245	245	D	this study
**Rufete**	132	156	222	232	239	257	177	185	191	197	243	245	A	[2]
**Samarrinho**	132	150	228	228	239	257	175	185	189	197	243	249	D	[2]
**Síria**	136	150	218	230	239	249	177	177	189	207	245	245	A	[2]
**Tamarez**	144	150	222	232	239	243	175	177	191	197	245	249	D	[2]
**Tempranillo**	142	144	232	232	239	253	179	179	199	203	245	249	A	[2,4]
**Tinta Aguiar**	142	144	222	228	239	243	185	191	195	197	243	255	D	[1]
**Tinta Barroca**	142	150	224	232	239	243	177	179	191	195	243	245	D	[4,6]
**Tinta Carvalha**	144	150	228	232	249	263	177	185	197	207	245	249	D	[4]
**Tinta Francisca**	132	132	234	236	239	239	181	185	189	201	241	245	A	[4]
**Tinta Mesquita**	132	144	222	228	239	263	185	191	191	199	243	255	D	[1]
**Tinto Cão**	132	132	228	230	239	263	177	181	189	197	245	249	A	[4,6]
**Touriga Fêmea**	142	142	232	236	239	257	175	185	191	197	243	249	A	[1]
**Touriga Franca**	142	150	222	224	239	243	177	179	195	197	243	245	D	[2,4,6]
**Touriga Nacional**	142	152	222	232	239	239	177	185	191	197	243	243	A	[4]
**Trincadeira**	132	150	230	234	239	249	177	181	191	207	245	249	D	[2,4]
**Trousseau Noir**	142	150	234	234	239	257	171	185	191	191	243	245	A	[2,7]
**Vinhão**	132	134	218	222	239	263	185	185	191	199	243	249	A	[4]
**NG001**	136	150	230	234	241	243	177	185	189	191	245	249	D	this study
**NG002**	136	158	222	232	239	239	177	185	189	189	245	249	D	this study
**NG003**	144	152	224	234	239	239	185	185	191	191	243	255	D	this study
**NG004**	142	158	222	224	241	243	179	185	191	195	245	249	D	this study
**NG005**	132	150	232	234	239	239	177	185	189	191	245	249	D	this study
**NG006**	132	144	224	230	239	263	177	191	191	197	245	255	D	this study
**NG007**	144	144	228	232	243	253	185	191	199	203	249	255	D	this study
**NG008**	142	144	224	232	239	239	181	181	189	191	245	249	D	this study
**NG009**	132	142	224	230	239	243	177	191	191	195	245	255	D	this study
**NG010**	142	150	222	232	239	239	177	185	191	197	241	249	D	this study
**NG011**	132	144	224	236	243	249	181	191	195	**205**	249	255	D	this study
**NG012**	132	144	224	230	**237**	239	177	185	191	201	249	255	D	this study
**NG013**	132	140	226	228	239	243	171	175	191	195	**247**	249	A	this study

[1] Specific SSR alleles (those which occurred in no more than one genotype) are bold underlined.
[2] Designation according to Arroyo-García *et al.* [8].

Table S4. List of the 65 non-redundant grape genotypes detected at 46 polymorphic SNP loci. Chlorotypes were determined using the combination of SNP_NG_C_001, SNP_NG_C_003 and SNP_NG_D_003 loci (**CCG** correspond to chl **A**, **CTG** to chl **B**, **TTG** to chl **C** and **CCA** to **D**).

Genotype	SNP1003_336	SNP1015_67	SNP1027_69	SNP1035_226	SNP1079_58	SNP1127_70	SNP1157_64	SNP1215_138	SNP1229_219	SNP1323_155	SNP1349_174	SNP1399_81	SNP1411_565	SNP1445_218	SNP1453_40
**Afus Ali**	AA	GG	CC	CT	AG	GG	TT	TT	CC	CC	AG	AA	TT	AG	AG
**Alfrocheiro**	AC	GG	CT	CT	AG	GT	AT	TT	CG	AA	AG	AA	AT	AG	AG
**Alvarelhão Ceitão**	AC	AG	CT	TT	AG	GG	TT	CT	CG	AC	GG	AA	TT	AG	GG
**Baga**	CC	AG	TT	CT	GG	GT	AT	CT	CG	CC	GG	AG	TT	AG	AG
**Barca**	AC	GG	CC	CC	GG	GT	AT	TT	GG	CC	GG	AA	AT	AA	AG
**Black Monukka**	AC	AG	CT	CT	AG	GT	AT	TT	CG	AC	GG	AA	TT	AG	AG
**Camarate Tinto**	AC	AG	CT	TT	AG	GG	AT	CT	CG	AC	AA	AA	TT	AA	AG
**Carrega Branco**	CC	AG	CC	CC	AG	GT	TT	CT	CC	CC	GG	AA	TT	AG	AG
**Carrega Tinto**	CC	AG	TT	TT	AG	GT	AT	CT	CG	AC	AG	AA	TT	GG	AG
**Casculho**	AC	AG	CC	CT	AA	GG	TT	CT	CG	AC	AA	AA	AT	AG	GG
**Castelão**	CC	AG	CT	CC	AA	GT	TT	CT	CG	AC	AA	AA	TT	AA	GG
**Chasselas**	AA	GG	CT	CT	AG	TT	TT	CT	CC	AC	AG	AA	TT	AG	AA
**Cidadelhe**	AC	GG	CC	CC	AG	GT	TT	CC	GG	AC	GG	AA	TT	AA	AA
**Cornifesto**	CC	AG	CT	TT	AA	GT	TT	CT	CG	AC	AG	AA	TT	AG	AG
**Dodrelyabi**	AC	GG	CT	TT	AG	GG	TT	TT	CG	CC	AA	AA	TT	AG	AA
**Donzelinho Roxo**	--	AG	CC	CC	AG	GT	TT	CT	CG	AC	AA	AA	AT	AA	AG
**Folha de Figueira**	AC	GG	TT	CT	AG	GG	TT	CT	CC	CC	AG	AA	AT	AG	AA
**Gouveio**	CC	AG	CC	CT	GG	GT	TT	CT	CC	AC	AG	AA	AT	AA	AG
**Grand Noir**	CC	AG	CC	CC	GG	GT	AT	TT	CG	AC	GG	AA	AA	AG	AA
**Grec Rouge**	CC	AG	CT	CT	GG	TT	TT	TT	CG	AC	AA	AA	AT	AG	GG
**Hebén**	CC	AG	TT	CT	AG	GG	TT	CC	CC	CC	GG	AA	TT	GG	AG
**Jeronimo**	CC	GG	CT	CT	AG	GG	TT	CT	CC	CC	AG	AA	TT	GG	AA
**Malandra**	CC	GG	CT	TT	GG	GT	TT	CT	CG	AC	GG	AA	TT	AG	AG
**Malvasia Fina**	CC	GG	TT	CT	AG	GG	AT	CT	CC	AC	GG	AA	TT	AG	AG
**Malvasia Preta**	AC	AG	CT	CT	AA	GT	AT	CT	CG	AC	AA	AA	TT	GG	AG
**Marufo**	CC	AG	CT	CT	AG	GG	TT	CT	GG	CC	AG	AA	AT	AA	AG
**Molar**	AC	AG	CT	CT	GG	GT	AT	TT	CG	AA	AG	AA	AT	AG	AG
**Montua**	AC	AG	CT	CT	AG	GG	TT	CC	CC	CC	AG	AA	TT	GG	AA
**Mouratón**	CC	AG	CC	CT	AG	GT	AT	CT	CG	AC	AG	AA	TT	AG	AG
**Mourisco de Semente**	AC	AG	CT	TT	GG	GT	AT	CC	CG	AC	GG	AA	AT	AG	AG
**Nevoeira**	AC	GG	CT	CT	AG	GT	TT	CC	CG	CC	AA	AA	TT	AA	AA
**Palomino Fino**	AC	GG	TT	TT	AG	GG	TT	CT	CC	CC	AG	AA	AT	GG	AG
**Parraleta**	AC	GG	CT	TT	GG	GG	TT	CT	CG	AA	AG	AA	AT	AA	AA
**Perlette**	AA	GG	CC	CC	AG	GG	AT	CC	CC	AC	AG	AA	AT	GG	AG

Table S4. (cont.)

Genotype	SNP1003_336	SNP1015_67	SNP1027_69	SNP1035_226	SNP1079_58	SNP1127_70	SNP1157_64	SNP1215_138	SNP1229_219	SNP1323_155	SNP1349_174	SNP1399_81	SNP1411_565	SNP1445_218	SNP1453_40
**Roseira**	CC	AG	CT	CT	GG	GG	TT	CT	GG	CC	GG	AA	TT	AG	AG
**Rufete**	AC	AG	CC	TT	AG	TT	TT	CT	CG	AA	AG	AA	TT	AG	AA
**Samarrinho**	AC	GG	CT	CT	AG	GT	AT	CT	CC	AC	AG	AG	TT	AG	AA
**Síria**	CC	AG	CC	TT	AG	GG	TT	CT	CC	CC	AG	AA	TT	AA	AG
**Tamarez**	CC	AG	TT	TT	AA	GG	TT	CT	CC	AA	AG	AA	TT	AG	AG
**Tempranillo**	CC	GG	CC	CT	AG	GG	TT	CC	CC	AC	AA	AA	AT	GG	GG
**Tinta Aguiar**	CC	GG	CT	CC	GG	GT	TT	TT	GG	CC	GG	AA	AA	AA	AG
**Tinta Barroca**	CC	AG	CT	CC	AG	GT	TT	TT	GG	CC	AG	AA	AT	AA	AG
**Tinta Carvalha**	CC	AG	CC	CT	AA	GT	TT	CT	CG	CC	AA	AA	TT	GG	AG
**Tinta Francisca**	CC	GG	CC	TT	AA	GT	AT	CT	CG	AC	GG	AA	AT	GG	AA
**Tinta Mesquita**	CC	AG	CT	TT	GG	GG	TT	CC	GG	AC	AG	AA	AT	AG	AA
**Tinto Cão**	AC	AG	CT	TT	AG	GG	TT	CC	CG	AC	AG	AG	AT	AG	AA
**Touriga Fêmea**	CC	GG	CT	CC	GG	GT	TT	TT	CG	CC	AG	AA	TT	AG	AG
**Touriga Franca**	AC	AG	CC	CC	GG	GT	TT	CT	GG	CC	AG	AA	AT	AA	AA
**Touriga Nacional**	AC	GG	CC	CT	GG	TT	AT	TT	CG	CC	AG	AA	AT	AG	AA
**Trincadeira**	CC	AG	CT	TT	AA	GG	TT	CC	CG	AC	AG	AA	TT	AG	AG
**Trousseau Noir**	AC	GG	CC	CT	GG	GT	AT	CT	CC	CC	AG	AG	TT	AG	AA
**Vinhão**	CC	GG	CT	CT	GG	GT	AT	CT	GG	AA	AG	AA	TT	AG	AG
**NG001**	AC	AA	CC	CT	AG	GT	TT	CT	CC	CC	AG	AA	TT	GG	GG
**NG002**	CC	AG	CC	CC	AG	GT	TT	CT	CC	AC	GG	AA	TT	AA	GG
**NG003**	CC	AG	CT	CT	GG	GT	TT	CT	CG	CC	AA	AG	TT	AG	AG
**NG004**	CC	AG	CT	CC	AG	GT	TT	CT	CG	CC	AG	AA	AT	AA	AG
**NG005**	AC	AA	CC	TT	AG	GT	TT	CT	CC	CC	AG	AA	TT	AG	AG
**NG006**	CC	AA	CT	TT	AG	GG	TT	CT	CG	CC	AG	AA	AT	AA	AA
**NG007**	CC	GG	CT	TT	GG	GG	AT	CT	GG	AC	AG	AA	AT	AA	AG
**NG008**	AC	AG	TT	CC	AA	GG	TT	TT	CG	CC	AA	AA	AT	AA	AG
**NG009**	CC	GG	CC	TT	AA	GG	TT	CT	GG	AC	AG	AA	AT	AA	AG
**NG010**	--	AG	--	CT	GG	--	TT	CT	GG	CC	--	AA	--	--	AG
**NG011**	CC	AA	CT	TT	AG	GG	TT	CT	CG	CC	AA	AA	AT	AG	AG
**NG012**	CC	AG	CT	CT	GG	GG	AT	CT	GG	AC	GG	AA	AT	AG	AG
**NG013**	AC	GG	CT	CC	AG	TT	TT	TT	CG	AC	AA	AA	AT	GG	AA

Table S4. (cont.)

Genotype	SNP1513_153	SNP191_100	SNP197_82	SNP259_199	SNP269_308	SNP325_65	SNP425_205	SNP447_244	SNP555_132	SNP579_187	SNP581_114	SNP593_149	SNP613_315	SNP697_296	SNP819_210
**Afus Ali**	TT	CC	AC	AA	AG	AT	AA	CT	AA	TT	AA	TT	CC	AA	AT
**Alfrocheiro**	TT	CC	AC	AA	AG	AA	AA	CC	AC	TT	GG	CT	CC	AA	AT
**Alvarelhão Ceitão**	CT	CC	AC	AT	GG	AT	AA	CT	AC	TT	GG	CT	TT	AG	AT
**Baga**	CT	CT	AC	AT	AG	AA	AA	CC	AC	CT	GG	TT	CC	AG	TT
**Barca**	CT	CT	CC	TT	GG	AA	AA	CT	AA	CT	GG	CT	CC	--	AT
**Black Monukka**	TT	CC	AC	AT	AA	AA	AA	CT	AC	TT	AG	TT	CT	AA	TT
**Camarate Tinto**	TT	CC	CC	AA	AG	AA	AA	CC	AC	CT	GG	TT	CC	AG	AT
**Carrega Branco**	CT	CC	CC	TT	GG	AA	AA	CT	AA	CT	AG	TT	TT	AG	AT
**Carrega Tinto**	TT	CC	CC	AT	GG	AT	AA	CC	AC	CT	GG	CT	CC	AA	AA
**Casculho**	TT	CC	CC	AT	GG	AA	AA	CC	AC	TT	AG	CT	CT	AG	AT
**Castelão**	TT	CC	AC	AT	GG	AA	AA	CC	AA	CT	GG	CT	CT	AG	AA
**Chasselas**	CC	CT	CC	AT	AA	AT	AA	CC	AC	TT	AG	CC	CT	AA	AT
**Cidadelhe**	TT	CC	CC	AT	AA	AA	AA	CC	AA	CT	GG	CC	CT	GG	AA
**Cornifesto**	TT	CC	CC	AT	AG	AA	AA	CC	AA	CT	AG	CT	CC	AG	AA
**Dodrelyabi**	TT	CC	CC	TT	AG	TT	AA	CT	AC	TT	AG	CT	CC	AA	TT
**Donzelinho Roxo**	TT	CC	CC	AT	AA	AA	AA	CT	AA	CC	GG	CT	CT	AG	AA
**Folha de Figueira**	CC	CC	AC	AA	GG	AT	AA	CT	AC	CT	AG	CT	CC	AA	AT
**Gouveio**	CT	CC	AC	AA	AG	AA	AA	CT	AA	CT	GG	TT	CC	AA	AT
**Grand Noir**	CC	CC	AC	TT	AG	AT	AA	CT	AA	TT	AG	TT	CT	AA	AA
**Grec Rouge**	CT	CC	CC	AT	AG	AT	AC	CT	AC	CT	AG	CC	CT	AA	AT
**Hebén**	TT	CC	CC	TT	GG	AT	AA	CC	AA	CT	GG	CT	CT	AG	AT
**Jeronimo**	TT	CC	CC	AT	AG	AT	AA	CT	AA	TT	AG	TT	CT	AG	TT
**Malandra**	CT	CC	AA	AT	AG	TT	AA	CC	AC	TT	AG	TT	CC	AA	TT
**Malvasia Fina**	TT	CC	CC	AT	AG	AA	AA	CC	AC	TT	GG	TT	CT	AA	TT
**Malvasia Preta**	TT	CC	CC	AT	AG	AA	AA	CC	AA	CT	GG	TT	CC	AG	AA
**Marufo**	CT	CC	CC	TT	AG	AA	AA	CT	AA	CT	GG	CT	CT	GG	AA
**Molar**	TT	CT	AA	AT	AG	AA	AA	TT	AA	TT	GG	CT	CC	AA	AT
**Montua**	TT	CC	CC	TT	GG	AA	AA	CC	AA	TT	AG	TT	CT	AG	AA
**Mouratón**	TT	CC	CC	AT	AG	AA	AA	CC	AC	CT	AG	CT	CT	AG	AA
**Mourisco de Semente**	TT	CC	CC	AT	AG	AA	AA	CC	AC	CT	GG	CC	CC	AG	AT
**Nevoeira**	CC	CC	AA	AT	AG	AT	AC	CT	AC	TT	AA	TT	CC	AA	TT
**Palomino Fino**	CT	CC	CC	TT	AG	AT	AC	CC	AA	CT	AG	CT	CT	AA	TT
**Parraleta**	CT	CC	CC	TT	AG	AT	AA	CC	AC	CT	AG	TT	CT	AA	AT
**Perlette**	CC	CC	CC	AA	AG	AA	AA	TT	AA	TT	AG	CT	CC	AA	AT

Table S4. (cont.)

Genotype	SNP1513_153	SNP191_100	SNP197_82	SNP259_199	SNP269_308	SNP325_65	SNP425_205	SNP447_244	SNP555_132	SNP579_187	SNP581_114	SNP593_149	SNP613_315	SNP697_296	SNP819_210
**Roseira**	TT	CC	AC	AT	AG	AA	AA	CC	AA	CT	GG	TT	TT	GG	AA
**Rufete**	CT	CC	AA	TT	AA	AA	AA	CT	AA	CT	AG	CT	CT	AG	AA
**Samarrinho**	CT	CC	AC	AT	AG	AA	AA	CT	AA	TT	GG	TT	CC	AA	AT
**Síria**	TT	CC	AC	AT	AG	AA	AA	CC	AC	CT	AG	TT	CT	AG	AT
**Tamarez**	TT	CC	CC	TT	AG	AA	AA	CC	AA	TT	GG	TT	TT	AG	AT
**Tempranillo**	CC	CC	AA	AA	GG	TT	AA	CC	AC	TT	AG	TT	CC	AA	AA
**Tinta Aguiar**	CT	CT	CC	TT	GG	AA	AA	CC	AA	CT	GG	CT	CC	AG	AT
**Tinta Barroca**	CC	CT	AC	TT	AG	AA	AA	CC	AA	TT	GG	CT	CT	AG	AT
**Tinta Carvalha**	CT	CT	CC	TT	AG	AA	AA	CT	AC	TT	AG	TT	CT	AG	AT
**Tinta Francisca**	CC	CC	AA	TT	AA	AA	AC	CC	AC	TT	AA	TT	CC	AA	TT
**Tinta Mesquita**	CC	CC	CC	TT	AA	AA	AA	TT	AC	TT	GG	CT	CT	AG	AT
**Tinto Cão**	CC	CC	CC	AT	AG	AA	AA	CC	AA	TT	AG	TT	CT	AG	AT
**Touriga Fêmea**	CT	CC	CC	TT	GG	AA	AA	CC	AA	TT	GG	TT	CT	AA	TT
**Touriga Franca**	CC	CC	AC	TT	AG	AA	AA	CT	AA	CT	GG	TT	CC	AG	AT
**Touriga Nacional**	CC	CT	AC	TT	AG	AA	AA	CC	AA	TT	GG	TT	CC	AA	TT
**Trincadeira**	TT	CC	AC	AT	AG	AT	AA	CC	AC	CT	AG	TT	TT	AG	TT
**Trousseau Noir**	CT	CC	AC	TT	AA	AA	AA	CT	AC	TT	GG	TT	CC	AA	AT
**Vinhão**	CT	CC	AC	TT	AG	AA	AA	CT	CC	TT	GG	CT	CC	AA	AT
**NG001**	CT	CT	AC	AA	GG	AA	AA	CC	AC	TT	AG	TT	CT	AG	AT
**NG002**	CC	CT	AC	AA	AG	AA	AA	CT	AA	TT	GG	TT	CC	AG	AT
**NG003**	CT	CC	AC	TT	AG	AA	AA	CT	AA	TT	GG	CT	CC	AG	AT
**NG004**	TT	CC	AC	AT	GG	AA	AA	CT	AA	CC	GG	TT	CT	AG	AA
**NG005**	CC	CT	AA	TT	AG	AA	AA	CC	AA	TT	AG	TT	CT	AA	AT
**NG006**	CC	CC	CC	AT	AA	AA	AA	CT	AA	TT	AG	TT	CC	GG	AT
**NG007**	TT	CC	CC	AT	AG	AA	AA	CC	AC	CT	GG	CT	CC	AG	AA
**NG008**	CC	CC	AC	AT	GG	AA	AA	TT	AC	TT	AG	CC	CC	AG	AT
**NG009**	CT	CC	CC	TT	GG	AT	AA	CT	AA	CT	AG	CT	TT	GG	AT
**NG010**	CT	CC	AC	AT	AG	AA	AA	--	AA	CT	GG	TT	--	--	--
**NG011**	CT	CC	CC	TT	GG	AA	AA	CT	AC	CC	AG	CT	CT	GG	AT
**NG012**	CC	CC	AC	TT	AA	AA	AA	CT	AC	CT	GG	CT	CT	AG	AA
**NG013**	CC	CC	AC	TT	AG	AT	AA	CT	AA	CT	GG	TT	CC	AA	AT

Table S4. (cont.)

Genotype	SNP829_281	SNP873_244	SNP879_308	SNP895_382	SNP945_88	SNP947_288	Vvi_10113	Vvi_10353	Vvi_10992	Vvi_12882	Vvi_1617	Vvi_9227	Vvi_9920	SNP_NG_C_001	SNP_NG_C_003	SNP_NG_D_003
**Afus Ali**	GG	CC	AG	AA	AG	AG	AA	GG	TT	CT	CC	AA	AA	C	C	G
**Alfrocheiro**	GG	CT	AA	AA	AA	AG	AG	GG	AT	CT	CC	TT	GG	C	C	G
**Alvarelhão Ceitão**	GG	CT	GG	TT	AG	GG	GG	GG	AA	TT	AA	AT	GG	C	C	G
**Baga**	GG	CC	AA	AT	AG	GG	AA	GG	TT	CT	AA	TT	GG	C	C	G
**Barca**	AG	CT	AA	AT	AG	GG	--	GG	AT	CC	AC	TT	GG	C	C	A
**Black Monukka**	AG	CT	AG	AT	AG	AA	AA	AA	AT	TT	CC	TT	GG	T	T	G
**Camarate Tinto**	GG	CC	AA	AA	AG	AG	AG	GG	AT	TT	AC	TT	GG	C	C	G
**Carrega Branco**	AG	CT	AA	AT	AG	GG	AG	AG	AT	TT	AC	AT	GG	C	C	A
**Carrega Tinto**	GG	CT	AA	AA	AA	AG	AG	GG	AT	TT	CC	AT	GG	C	C	G
**Casculho**	GG	CC	AG	AT	AG	AG	GG	GG	AT	CT	AC	AT	GG	C	C	G
**Castelão**	GG	CC	AG	AA	AG	AA	AA	GG	AT	TT	CC	TT	GG	C	C	G
**Chasselas**	AA	TT	AA	AT	AG	AG	AA	GG	AA	TT	AA	TT	AG	C	C	A
**Cidadelhe**	GG	CC	AA	TT	GG	AG	GG	GG	TT	TT	AC	TT	GG	C	C	A
**Cornifesto**	GG	CC	AA	AT	AG	AA	AG	GG	AT	TT	CC	AT	GG	C	C	G
**Dodrelyabi**	AA	CT	GG	AT	AG	GG	AA	GG	AA	TT	AC	TT	AG	C	T	G
**Donzelinho Roxo**	GG	TT	AA	AA	AG	AG	GG	GG	AA	CT	AA	TT	GG	C	C	A
**Folha de Figueira**	AA	CC	AG	AT	AG	GG	AG	GG	AA	TT	AC	AA	GG	C	C	G
**Gouveio**	AG	TT	AA	AT	AG	AA	GG	GG	AA	TT	AC	AT	GG	C	C	G
**Grand Noir**	AG	TT	AG	AA	AA	GG	AA	GG	AT	CC	AC	TT	AG	C	C	G
**Grec Rouge**	AA	TT	AA	AA	AA	GG	AG	GG	AA	TT	AC	AA	GG	C	C	G
**Hebén**	AG	CT	AG	AT	AG	AG	AG	GG	AT	TT	AC	AT	GG	C	C	G
**Jeronimo**	GG	CT	AG	AA	AG	AG	AG	GG	AA	TT	AA	AT	GG	C	C	G
**Malandra**	AG	CT	GG	AT	AG	AA	AG	GG	TT	TT	AC	TT	AG	C	C	A
**Malvasia Fina**	GG	CT	AG	AT	AG	GG	AA	GG	TT	CT	AC	TT	GG	C	C	G
**Malvasia Preta**	GG	CT	AA	AT	AG	AG	AG	GG	AT	TT	AC	TT	GG	C	C	G
**Marufo**	GG	CT	AA	AT	GG	GG	AG	GG	AT	CT	AC	TT	AG	C	C	A
**Molar**	GG	CT	AG	AA	AA	AG	AA	GG	AA	CT	CC	TT	GG	C	C	G
**Montua**	AG	CT	AA	AT	AA	AA	AA	GG	AT	TT	AC	AT	AG	C	C	G
**Mouratón**	GG	CC	AA	AT	AG	GG	GG	GG	TT	CT	AC	AT	GG	C	C	G
**Mourisco de Semente**	AG	CT	AG	TT	AG	GG	AG	AG	AA	TT	AA	TT	GG	C	C	A
**Nevoeira**	AA	CC	GG	TT	AG	AG	GG	AG	AA	TT	AC	AT	GG	C	C	G
**Palomino Fino**	AG	TT	GG	AT	AG	AG	AG	GG	AT	TT	AC	AT	GG	C	C	A
**Parraleta**	AG	CC	AG	AT	AG	AG	AA	GG	AT	CT	AC	AT	GG	C	C	G
**Perlette**	GG	CC	AA	AT	AA	GG	AA	GG	TT	TT	AC	TT	GG	C	C	G

Table S4. (cont.)

Genotype	SNP829_281	SNP873_244	SNP879_308	SNP895_382	SNP945_88	SNP947_288	Vvi_10113	Vvi_10353	Vvi_10992	Vvi_12882	Vvi_1617	Vvi_9227	Vvi_9920	SNP_NG_C_001	SNP_NG_C_003	SNP_NG_D_003
**Roseira**	GG	CC	AA	AA	AG	AG	GG	GG	AT	CT	AC	TT	AG	C	C	A
**Rufete**	GG	CT	AG	AT	AA	AG	AG	AG	AT	CT	CC	AT	GG	C	C	G
**Samarrinho**	AG	CT	AG	TT	AA	AG	AA	GG	AT	CT	CC	TT	GG	C	C	A
**Síria**	AG	CC	AG	TT	AG	GG	AA	GG	AT	TT	CC	AT	GG	C	C	G
**Tamarez**	GG	CT	AG	TT	GG	AG	AA	AG	AT	TT	AC	AT	GG	C	C	A
**Tempranillo**	AA	TT	AA	AA	AG	AG	AA	GG	TT	TT	AC	TT	AA	C	C	G
**Tinta Aguiar**	AG	--	AA	AT	AG	GG	AG	GG	AT	CC	AC	TT	AG	C	C	A
**Tinta Barroca**	AG	CC	AA	AA	AG	AG	AA	GG	AT	CT	AC	TT	GG	C	C	A
**Tinta Carvalha**	GG	CC	AG	AT	GG	GG	GG	GG	AT	TT	AC	AT	GG	C	C	A
**Tinta Francisca**	AG	CT	AA	AT	AG	AA	AG	AG	AT	TT	AC	AT	AG	C	C	G
**Tinta Mesquita**	AG	CT	AG	AT	AG	GG	GG	GG	AT	TT	AC	TT	GG	C	C	A
**Tinto Cão**	AG	CC	AG	TT	AG	--	AA	AG	AT	TT	CC	AT	GG	C	C	G
**Touriga Fêmea**	AG	--	AG	AT	AA	AG	AA	GG	AT	CC	AA	TT	GG	C	C	G
**Touriga Franca**	AG	CT	AA	TT	AG	AG	AA	GG	AA	CT	AA	TT	AG	C	C	A
**Touriga Nacional**	AA	--	AA	AT	AA	--	AA	GG	AT	CT	AC	TT	GG	C	C	G
**Trincadeira**	AA	CC	AG	AT	AA	GG	AA	GG	TT	TT	AC	AT	GG	C	C	A
**Trousseau Noir**	GG	CC	AG	AT	AG	GG	AA	AG	AA	CC	AC	AT	GG	C	C	G
**Vinhão**	AA	CT	AG	TT	AA	GG	AG	AG	AA	TT	CC	TT	AG	C	C	G
**NG001**	GG	CT	AG	AT	AG	GG	AG	GG	TT	CT	AC	TT	GG	C	C	A
**NG002**	AG	CT	AA	TT	AG	AG	AG	GG	AT	TT	CC	TT	GG	C	C	A
**NG003**	GG	CT	AG	AT	GG	GG	AG	AG	AT	CT	AC	TT	AG	C	C	A
**NG004**	GG	TT	AA	AT	AG	AG	AG	GG	AT	TT	AC	TT	AG	C	C	A
**NG005**	GG	CT	AG	AA	AG	GG	GG	GG	AT	CT	CC	AA	AG	C	C	A
**NG006**	AG	CT	AG	AT	GG	GG	AG	AG	AT	CT	CC	AT	AG	C	C	A
**NG007**	GG	CC	AA	AT	GG	GG	GG	GG	AT	TT	AC	TT	AG	C	C	A
**NG008**	AG	CT	AA	AA	GG	GG	AA	GG	AT	CT	AC	AT	GG	C	C	A
**NG009**	AG	CC	AG	TT	AG	GG	AG	GG	AT	CT	AC	AT	AG	C	C	A
**NG010**	--	--	AA	AT	AG	AG	AG	GG	AT	CT	AC	TT	AG	C	C	A
**NG011**	AG	CC	AA	TT	AG	GG	AG	GG	TT	CT	AA	AT	GG	C	C	A
**NG012**	AG	CT	AG	AT	AG	GG	GG	AG	AA	CT	CC	TT	AA	C	C	A
**NG013**	AA	CT	AG	AA	AA	GG	AA	AG	TT	CT	CC	AT	GG	C	C	G

Table S5. Genetic parameters, and allele sizes and frequencies over 6 microsatellite loci in the 65 non-redundant genotypes analysed in this study.

SSR markers	**VVS2**		**VVMD5**		**VVMD7**		**VVMD27**		**VrZAG62**		**VrZAG79**	
Na	12		9		10		7		9		10	
Ne	5.545		6.995		3.928		5.027		4.120		4.154	
Ho	0.892		0.954		0.785		0.877		0.785		0.862	
He	0.820		0.857		0.745		0.801		0.757		0.759	
PIC	0.793		0.844		0.736		0.777		0.738		0.749	
Allele no.	***AS***	***AF***	***AS***	***AF***	***AS***	***AF***	***AS***	***AF***	***AS***	***AF***	***AS***	***AF***
1	132	0.185	218	0.023	**237**	**0.008**	171	0.015	189	0.123	**237**	**0.008**
2	134	0.038	222	0.169	239	0.438	175	0.115	191	0.431	241	0.031
3	136	0.046	224	0.108	241	0.015	177	0.262	195	0.077	243	0.115
4	**138**	**0.008**	226	0.015	243	0.177	179	0.062	197	0.138	245	0.300
5	140	0.015	228	0.115	247	0.031	181	0.123	199	0.031	**247**	**0.008**
6	142	0.262	230	0.138	249	0.077	185	0.277	201	0.015	249	0.331
7	144	0.162	232	0.177	253	0.123	191	0.146	203	0.085	**253**	**0.008**
8	150	0.215	234	0.169	**255**	**0.008**			**205**	**0.008**	255	0.162
9	152	0.015	236	0.085	257	0.085			207	0.092	257	0.015
10	**154**	**0.008**			263	0.038					259	0.023
11	156	0.031										
12	158	0.015										

Na - Average number of different alleles per locus; *Ne* - number of effective alleles; *Ho* - observed heterozygosity; *He* - expected heterozygosity; *PIC* - polymorphism information content.
AS - Allele sizes, in base pairs; *AF* - allele frequencies (AF).
Unique alleles show an *AF* = 0.08 and they are bold underlined (see **Supplementary table S3**).

Table S6. Structure results at K = 2 based on 226 SNP markers. Genotypes with membership coefficients (*q* - values) below the threshold of 0.7 for genetic group assignment were admixed.

	q-value		Estimated genetic group
Prime name	SNP-group 1	SNP-group 2	
Afus Ali	0.455	0.545	Admixed
Alfrocheiro	0.982	0.018	1
Alvarelhão Ceitão	0.648	0.352	Admixed
Baga	0.767	0.233	1
Barca	0.019	0.981	2
Black Monukka	0.582	0.418	Admixed
Camarate Tinto	0.997	0.003	1
Carrega Branco	0.702	0.298	1
Carrega Tinto	0.994	0.006	1
Casculho	0.980	0.020	1
Castelão	0.996	0.004	1
Chasselas	0.243	0.757	2
Cidadelhe	0.510	0.490	Admixed
Cornifesto	0.995	0.005	1
Dodrelyabi	0.523	0.477	Admixed
Donzelinho Roxo	0.301	0.699	2
Folha de Figueira	0.403	0.597	Admixed
Gouveio	0.529	0.471	Admixed
Grand Noir	0.514	0.486	Admixed
Grec Rouge	0.405	0.595	Admixed
Hebén	0.726	0.274	1
Jerónimo	0.532	0.468	Admixed
Malandra	0.632	0.368	Admixed
Malvasia Fina	0.994	0.006	1
Malvasia Preta	0.997	0.003	1
Marufo	0.018	0.982	2
Molar	0.735	0.265	1
Montua	0.636	0.364	Admixed
Mouratón	0.996	0.004	1
Mourisco de Semente	0.258	0.742	2
Nevoeira	0.556	0.444	Admixed
Palomino Fino	0.532	0.468	Admixed

Table S6. (cont.)

Prime name	*q*-value		Estimated genetic group
	SNP-group 1	SNP-group 2	
Parraleta	0.606	0.394	Admixed
Perlette	0.582	0.418	Admixed
Roseira	0.535	0.465	Admixed
Rufete	0.768	0.232	1
Samarrinho	0.632	0.368	Admixed
Síria	0.835	0.165	1
Tamarez	0.840	0.160	1
Tempranillo	0.510	0.490	Admixed
Tinta Aguiar	0.007	0.993	2
Tinta Barroca	0.025	0.975	2
Tinta Carvalha	0.689	0.311	Admixed
Tinta Francisca	0.548	0.452	Admixed
Tinta Mesquita	0.135	0.865	2
Tinto Cão	0.379	0.621	Admixed
Touriga Fêmea	0.503	0.497	Admixed
Touriga Franca	0.005	0.995	2
Touriga Nacional	0.204	0.796	2
Trincadeira	0.962	0.038	1
Trousseau Noir	0.633	0.367	Admixed
Vinhão	0.336	0.664	Admixed
NG001	0.957	0.043	1
NG002	0.636	0.364	Admixed
NG003	0.388	0.612	Admixed
NG004	0.205	0.795	2
NG005	0.595	0.405	Admixed
NG006	0.347	0.653	Admixed
NG007	0.709	0.291	1
NG008	0.015	0.985	2
NG009	0.318	0.682	Admixed
NG010	0.158	0.842	2
NG011	0.292	0.708	2
NG012	0.268	0.732	2
NG013	0.634	0.366	Admixed

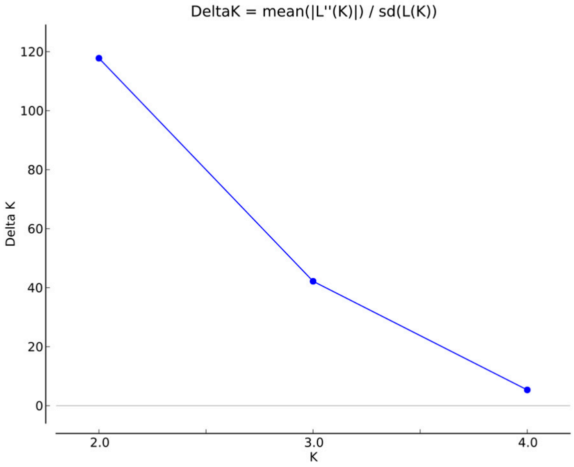

Figure S1. Delta K plots obtained from STRUCTURE HARVESTER to set the most likely number of genetic groups within the 65 non-redundant grape population identified in the present study, based on 226 SNP data.

References

1. Maul, E.; Töpfer, R.; Röckel, F.; Brühl, U.; Hundemer, M.; Mahler-Ries, A.; Walk, M.; Kecke, S.; Wolck, A.; Ganesch, A. Vitis International Variety Catalogue. 2021. Available online: www.vivc.de (accessed on 2 July 2021).
2. Cunha, J.; Teixeira-Santos, M.; Carneiro, l.C.; Fevereiro, P.; Eiras-Dias, J.E. Portuguese traditional grapevine cultivars and wild vines (*Vitis vinifera* L.) share morphological and genetic traits. *Genet. Res. Crop Evol.* **2009**, *56*, 975–989, https://doi.org/10.1007/s10722-009-9416-4.
3. Imazio, S.; Labra, M.; Grassi, F.; Scienza, A.; Failla, O. Chloroplast microsatellites to investigate the origin of grapevine. *Genet. Resour. Crop. Evol.* **2006**, *10*, 1–9, https://doi.org/10.1007/s10722-004-6896-0.
4. Castro, I.; Pinto-Carnide, O.; Ortiz, J.M.; Martín, J.P. Chloroplast genome diversity in Portuguese grapevine (*Vitis vinifera* L.) cultivars. *Mol. Biotechnol.* **2013**, *54*, 528–540, https://doi.org/10.1007/s12033-012-9593-9.
5. Augusto, D.; Oliveira, A.A.; Falco, V.; Castro, I. Uncovering Northeast Portugal grapevine's varietal legacy. *Vitis* **2019**, *58*, 89–93, https://doi.org/10.5073/vitis.2019.58.special-issue.89-93.
6. Baleiras-Couto, M.M.; Eiras-Dias, J.E. Detection and identification of grape varieties in must and wine using nuclear and chloroplast microsatellite markers. *Anal. Chim. Acta* **2006**, *563*, 283–291, https://doi.org/10.1016/j.aca.2005.09.076.
7. Moita Maçanita, A.; Santos, R.; Gomes, A.C.; Santos, A. Unravelling the origin of *Vitis vinifera* L. Verdelho. *Aust. J. Grape Wine Res.* **2018**, *24*, 450–460, https://doi.org/10.1111/ajgw.12353.
8. Arroyo-García, R.; LeFort, F.; de Andrés, M.T.; Ibáñez, J.; Borrego, J.; Jouve, N.; Cabello, F.; Martínez-Zapater, J.M. Chloroplast microsatellite polymorphisms in *Vitis* species. *Genome* **2002**, *45*, 1142–1149, https://doi.org/10.1139/g02-087.
